# Lipid reprogramming of stratified squamous epithelium by the high-risk HPV E6 and E6/E7 oncoproteins

**DOI:** 10.1007/s11306-026-02498-2

**Published:** 2026-07-01

**Authors:** Megan E. Spurgeon, Taylor E. Lange, Alexandra Baty, Helena Li, Paul F. Lambert, Kenneth D. R. Setchell, Susanne I. Wells, Xueheng Zhao

**Affiliations:** 1 John W. and Jeanne M. Rowe Center for Research in Virology, Morgridge Institute for Research, Madison, WI USA; 2 Department of Oncology/McArdle Laboratory for Cancer Research, University of Wisconsin School of Medicine and Public Health, Madison, WI USA; 3 Division of Oncology, Cincinnati Children’s Hospital Medical Center, Cincinnati, OH USA; 4 Division of Pathology and Laboratory Medicine, Cincinnati Children’s Hospital Medical Center, Cincinnati, OH USA; 5 Division of Biomedical Informatics, Cincinnati Children’s Hospital Medical Center, Cincinnati, OH USA; 6 Departmen of Pediatrics, University of Cincinnati College of Medicine, Cincinnati, OH USA

**Keywords:** High-risk human papillomavirus, Lipidomics, Biomarker, Oncoproteins

## Abstract

**Introduction:**

High risk human papillomavirus (HPV) infection and genome integration with pronounced expression of the viral E6/E7 oncogenes is the major cause of cervical cancer. Emerging evidence suggests that HPV reprograms host metabolism to support viral persistence and cellular transformation. However, global HPV oncogene-induced lipidomic reprogramming remains poorly understood, particularly at early stages of HPV-induced transformation.

**Objective:**

We sought to define the regulation of lipid metabolism in squamous epithelia of transgenic mice expressing the HPV16 oncogene E6 alone or in conjunction with E7.

**Methods:**

Untargeted lipidomics was used to identify novel lipid biomarkers in the skin and female reproductive tract (FRT) of HPV16 E6 and E6/E7 transgenic compared to wild-type (WT) mice. To investigate enzymatic dysregulation of lipids by HPV oncogene expression, we employed Lipid Network Explorer (LINEX^2^), which analyzes lipidomics data through lipid enrichment analysis. We also used the Global Natural Product Social Molecular Networking (GNPS) platform to enhance lipid identification, exploring molecular networking to improve feature annotation.

**Results:**

Our lipidomic analysis produced several new observations. First, E6 expression caused a consistent alteration of glycerophospholipids, with particularly significant substrate-product shifts in the phosphatidylcholine (PC) to lysophosphatidylcholine (LPC) pathway in the skin. Second, E6/E7 expression caused a dysregulation of glucosylceramide (GlcCer) biosynthesis. Third, both E6/E7 expressing skin and FRT tissues exhibited a redox imbalance and increased levels of oxidized lipids, including oxylipins and several oxidized PCs. These findings suggest that HPV oncoproteins drive lipid reprogramming, potentially contributing to early HPV-related tumorigenesis.

**Conclusions:**

These findings provide new insights into HPV‑induced lipid reprogramming and establish a framework for future studies examining the functional and clinical relevance of lipid alterations in HPV‑associated cancers.

**Supplementary Information:**

The online version contains supplementary material available at https://doi.org/10.1007/s11306-026-02498-2.

## Introduction

High-risk human papillomavirus (hrHPV) is the leading cause of virus-associated cancers and cancer-related mortality worldwide, affecting both women and men (Jensen et al., 2024). Globally, hrHPV accounts for approximately 5% of all human cancers (de Martel et al., 2017; McLaughlin-Drubin et al., 2008). In the United States alone, an estimated 80 million individuals are currently infected, with over 14 million new cases reported annually (Viens et al., 2016). Persistent HPV infection is a major driver of cervical cancer, one of the most prevalent and deadly cancers among women, as well as head and neck cancers and other anogenital malignancies (zur Hausen, 2002). Despite its widespread impact, there is currently no cure or fully effective antiviral therapy to eliminate HPV infection.

HPV is a double-stranded DNA virus with a circular genome of approximately 8,000 base pairs. Upon infecting epithelial cells, HPV exploits the host’s replication machinery to amplify its genome and produce progeny virions that spread to adjacent cells. Among the hrHPV types, HPV16 and HPV18 are responsible for the majority of HPV-associated cancers. These viruses induce cellular changes that progress from precancerous lesions to invasive cancer (Tota et al., 2011). A hallmark of HPV-driven carcinogenesis is the sustained overexpression of the viral oncoproteins E6 and E7, which promote oncogenesis by targeting and inactivating key tumor suppressors such as TP53 and members of the retinoblastoma protein family Rb, p107 and p130 (Engeland, 2022; Munger et al., 1989; Pal et al., 2019; Scheffner et al., 1990). These tumor suppressors are also crucial regulators of cellular metabolism.

Reprogramming of lipid metabolism has emerged as a hallmark of cancer (Cheng et al., 2018). Lipids are fundamental components of cellular membranes, essential for maintaining epithelial barrier function, organelle integrity, and signal transduction (Ikenouchi, 2018; Jungersted et al., 2008; Santos et al., 2018; Sunshine et al., 2017). Genes and proteins involved in lipid metabolism and signaling, including the PI3K/AKT/mTOR pathway, have been implicated in HPV-mediated carcinogenesis (Bossler et al., 2019; Danolic et al., 2020; Zhang et al., 2015). A recent study analyzing cervicovaginal lavages and vaginal swabs from HPV-negative and HPV-positive individuals, as well as women with varying grades of cervical dysplasia and cancer, linked lipid alterations to genital inflammation (Ilhan et al., 2019). Despite the recognized importance of lipids in hrHPV infection and cancer progression, a comprehensive understanding of how hrHPV oncoprotein regulate lipid metabolism in epithelial tissue has yet to be established.

In this study, we investigated lipid reprogramming driven by the HPV16 E6 and E7 oncoproteins in skin and mucosal epithelial tissues to advance knowledge of virus–host interactions relevant to carcinogenesis.

Lipidomics workflows encompass sample preparation, lipid extraction, chromatographic peak alignment, molecular ion feature identification, and biological interpretation. Among these steps, feature identification and biological interpretation remain challenging due to limited databases of experimentally validated tandem MS spectra and the lack of lipid-centric pathway analysis tools.

Recent bioinformatics advances have begun to address these limitations. Tools such as Global Natural Product Social Molecular Networking (GNPS) facilitate the discovery of novel lipids by constructing molecular networks based on MS/MS fragmentation similarities (Wang et al., 2016), enabling the identification of structurally related lipid species. Network-based approaches are particularly valuable given the vast diversity of lipid species and subclasses. The Lipid Network Explorer (LINEX^2^) is a recently developed tool that integrates lipid classes and metabolic reactions into a network framework, allowing for comprehensive interpretation of lipidomic changes (Rose et al., 2023).

Using transgenic mouse models expressing either HPV16 E6 alone or both E6 and E7, previously established for studies of HPV-mediated carcinogenesis (Spurgeon, 2022), we profiled lipidomes in skin and female reproductive tract (FRT) tissues prior to high-grade dysplasia or cancer development. We identified differentially abundant lipids and applied GNPS for enhanced lipid annotation via molecular networking. LINEX^2^ was then used to biologically interpret lipidomic data and assess enzymatic dysregulation. Our findings reveal that HPV16 E6/E7 and E6 oncoproteins modulate distinct lipid metabolic pathways in skin and FRT tissues, including sphingolipid metabolism and the phospholipid remodeling pathway. These methodological advances provide new insights into HPV-driven lipid reprogramming and lay the groundwork for identifying lipid biomarkers and therapeutic targets to detect and prevent HPV-induced transformation through metabolic intervention.

## Materials and methods

### Transgenic mice

*K14E6* and *K14E6/E7* transgenic mice were maintained on the *FVB/N* genetic background have been described previously (Herber et al., 1996; Song et al., 2000). Briefly, these mice carry transgenes encoding the E6 (*K14E6)* or E6 and E7 (*K14E6/E7*) proteins from the high-risk, cancer-associated HPV16 virus type. Transgenes are driven by the human keratin 14 promoter, leading to constitutive expression of E6 or E6/E7 in the basal keratinocytes of the stratified squamous epithelia of the ear, female reproductive and other tissues. In this study, all *K14E6* mice were heterozygous and all *K14E6/E7* mice were heterozygous for both alleles. Wild-type *FVB/N* mice were included as controls. Mice were housed and treated in American Association of Laboratory Animal Care-approved vivaria of the University of Wisconsin School of Medicine and Public Health (Madison, WI) according to a protocol approved by the University of Wisconsin Institutional Animal Care and Use Committee.

### Reagents

Lipid standards were purchased from Avanti Polar Lipids (Alabaster, AL) and Sigma-Aldrich (St Louis, MO). All solvents were of mass spectrometry grade and deionized water was obtained from a Milli-Q water system (Millipore, Milford, MA). VWR 2 mL × 2.8 mm ceramic hard tissue homogenizing mix (RNase & DNase free, Cat. No. 10158-612) pre-filled bead tubes was obtained from VMR (Phillipsburg, NJ).

### Sample preparation

To collect tissue for lipidomics analysis, mice were euthanized using an approved method of carbon dioxide asphyxiation. Entire ear or female reproductive tract tissues were immediately collected upon necropsy (Table 1). Tissues were placed in cryovials, snap frozen in liquid nitrogen, and stored at – 80 ºC until sample preparation. Tissue samples were weighed and then homogenized using a Precellys® Evolution tissue homogenizer (Bertin Technologies, Montigny-le-Bretonneux, France). The homogenate was extracted by chloroform:methanol:water (1:2:0.7, v/v/v) twice with solvent volume normalization by tissue weight to maintain a consistent mass to solvent ratio across all extractions (e.g. 1 mg tissue with 20 μL homogenization solvent) and obtain comprehensive lipid recovery. An aliquot of 20 µl of chilled methanol containing an internal standard cocktail including PE(17:0/17:0), PG(18:0/16:0)-d5, PC(18:1/16:0)-d31, Spingosine(17:0), Ceramide(d18:1/17:0); SM(d18:1/17:0); Palmitic acid-d3; Cholesterol-d7; TG(17:0/17:1/17:0)-d5; DG(12:0/12:0/0:0); MG(17:0/0:0/0:0); LPS(17:0), LPE(17:1), was added to each sample during extraction. These internal standards were included to monitor extraction efficiency, retention‑time stability, and instrument performance across the run. Importantly, internal‑standard peak areas were not used for data normalization. Lipid intensities were instead normalized using tissue weight measured before the extraction, ensuring comparability across samples. Internal standards therefore served exclusively as QC markers to assess batch stability, detect signal drift, and verify data quality throughout the acquisition. Sample extract was dried under nitrogen at 30 °C. Dried residue was reconstituted in 200 µL solvent consisting of isopropanol/acetonitrile/water (45:35:20, v/v/v) with 10 mM ammonium formate and 0.1% formic acid and centrifuged for 8 min at 4,000 rpm. The supernatant was then transferred to HPLC auto-sampler vials for UHPLC-HRMS analysis.

### Lipidomics analysis

The untargeted lipidomics analysis was conducted on a Q Exactive™ plus hybrid quadrupole-Orbitrap™ mass spectrometer interfaced with Vanquish ultra-high performance liquid chromatography (UHPLC) system (Thermo Scientific, Waltham, MA). A gradient mobile phase was used with a binary solvent system, which changed from 60% solvent A to 57% solvent A over 2 min, then to 50% solvent A at 2.1 min, then to 46% solvent A over 9.9 min, and then, after change to 30% at 12.1 min, to 1% solvent A over 5.9 min, then to 60% solvent A at 18.1 min and this was held for 2 min. Total run time was 20 min, and the flow rate was 0.4 mL/min. Solvent A consisted of acetonitrile/water (60/40) with 10 mM ammonium formate and 0.1% formic acid; solvent B consisted of isopropanol/acetonitrile (90/10) with 10 mM ammonium formate and 0.1% formic acid. The injection volume was 5 μL for both negative and positive ion mode. An Acquity CSH C18 UPLC column (2.1 × 100 mm, 1.7 µm, Waters, Milford, MA) was used for separation. Column temperature was set at 55 °C. The ESI source was operated in the following parameters: spray voltage is 2.5 kV, capillary temperature, 350 °C; sheath gas flow rate, 35; auxiliary gas heater temperature, 325 °C. Data were acquired using a full MS scan (m/z 150–1500; AGC target 3 × 10⁶; maximum injection time 100 ms; Orbitrap resolution 140,000) followed by collision‑induced dissociation–based data‑dependent MS/MS (Orbitrap resolution 17,500; AGC target 1 × 10^5^; maximum injection time 50 ms; loop count 15; top‑15 method; quadrupole isolation window 1.0 m/z; stepped NCE 20, 40, 60). Although the precursor ions were isolated using a 1.0 m/z window, all mass measurements were performed in the Orbitrap at high resolution, enabling accurate precursor and fragment mass determination. Data quality and instrument performance was monitored throughout the data acquisition using quality control (internal standards), method blanks and pooled samples. Data was processed using the Progenesis QI Analysis software (Waters, Milford, MA). Intensity values are representative of peak area. Complex lipids were identified by searching against a precursor accurate mass, retention time, in conjunction with matching tandem mass spectra library (in-house) as well as public spectral libraries including LIPID MAPS structure database (LMSD) and HMDB. Deconvolution, peak alignment, and preliminary normalization were conducted on raw metabolomics data with Progenesis QI™ (version 3.1). Default method parameters were used, except only [M + H]^+^, [M + Na]^+^, [M + K]^+^, [M + H–H_2_O]^+^, and [M + NH_4_]^+^ precursors were considered for positive ion mode analysis, and only [M − H]^−^, [M + Cl]^−^, and [M + HAc − H]^−^ precursors were considered for negative ion mode analysis. Each compound ion feature, i.e. deconvoluted peak in the mass chromatogram, is annotated by elution time with *m/z* ratio. Lipid annotation was conducted with public metabolome databases or in-house lipid curation libraries using accurate mass-to-charge ratio (m/z) in combination with retention time (RT) and tandem MS spectra. Lipid species annotation was achieved at either Metabolomics Standards Initiative (MSI) level 3 (putative annotation with lipid class and sum composition) or level 2 (putative identification with accurate mass and retention time matches a reference spectrum from our in-house database). Raw data (peak area) of each sample were normalized first by total compound ion intensity by Progrenesis QI™ before being normalized by tissue weight. If a lipid species were identified in both positive and negative ion mode acquisition, only dominant ion mode was used in the final data analysis. Putative marker metabolites were confirmed using authenticated standards if possible.

### Bioinformatics network analysis

To investigate lipid structural relationships and metabolic pathways, we employed two bioinformatics platforms: Global Natural Products Social Molecular Networking (GNPS) and Lipid Network Explorer (LINEX^2^). Raw MS data were first converted into mzML format and processed through the GNPS workflow for molecular networking. The data were filtered by removing all MS/MS fragment ions within ± 17 Da of the precursor m/z. Spectra were further refined by retaining only the top six fragment ions within a ± 50 Da window across the spectrum. The precursor ion mass tolerance was set to 2.0 Da, and the MS/MS fragment ion tolerance to 0.5 Da. A single adduct form was selected per compound. Discrepancies between Progenesis QI™ and GNPS alignments were resolved by using Progenesis QI™ as the primary feature-alignment framework and harmonizing GNPS features through ppm‑level m/z matching, retention‑time tolerance, adduct reconciliation, and MS/MS spectral confirmation, with ambiguous matches excluded to ensure consistent lipid feature identity. In summary, Progenesis QI™ for final feature alignment and quantitation, while GNPS was used for additional structural annotation and spectral confirmation.

A molecular network was constructed where edges were retained only if they had a cosine similarity score above 0.6 and more than six matched peaks. Additionally, edges were preserved only if both nodes appeared in each other’s top 10 most similar nodes. To manage network complexity, the maximum molecular family size was capped at 100 nodes, with the lowest scoring edges removed as needed. The resulting GNPS network was visualized using Cytoscape, allowing inference of structural similarity between known and unknown compounds. Manual review of lipid identifications was also conducted for quality control. Special attention was given to uncertain features, particularly oxidized or modified lipids, to support novel lipid identification and improve lipidome coverage.

For metabolic network analysis, LINEX^2^ was used to construct a global lipid network de novo, based on qualitative and quantitative associations among lipid species in the dataset (Rose et al., 2023). Prior to analysis, lipid nomenclature was verified using MetaboAnalyst 6.0 (Pang et al., 2024) and LipidLynxX (Ni et al., 2020). LINEX^2^ employs a local search algorithm that iteratively explores candidate solutions through node insertion, deletion, and substitution, optimizing for biologically meaningful subgraphs. From these re-constructed subgraphs, a list of target lipids was generated and subjected to enrichment analysis using LION/web (Molenaar et al., 2019), enabling deeper insight into the functional and biological implications of lipid alterations.

### Statistical analysis

For untargeted lipidomics analysis, the identification of lipid features significantly altered between genotypes (i.e., E6 vs. WT and E6/E7 vs. WT) was performed using univariate statistical methods. Significance was assessed based on both fold change and p-values. Multiple testing correction was performed using the Benjamini–Hochberg procedure to control the False Discovery Rate (FDR), with a threshold of adjusted p-value < 0.05 to control the family-wise error rate for analysis of cutaneous samples. Given the substantial heterogeneity of the female reproductive tissue samples, we used unadjusted p‑values for this dataset, in contrast to the skin dataset where FDR‑adjusted p‑values were applied. This approach was chosen to avoid overly conservative correction that could obscure biologically relevant signals. To explore functional patterns, lipid set enrichment analysis (LSEA) was conducted using the R packages “mdgsa” and “lipidr” (Mohamed et al., 2020), with the logarithm of the odds ratio (LOR) used to quantify enrichment of lipid subclasses. All statistical analyses were performed using the R programming language and environment for statistical computing (Team, 2021).

## Results

### Lipidomics profiling of cutaneous tissues from HPV16 transgenic mice

Lipidomics analysis of cutaneous (ear skin) and mucosal (female reproductive tract (FRT)) tissues from HPV oncoprotein-positive and WT mice identified over 1,000 lipid molecular species using both positive and negative ionization modes. To reduce redundancy in lipid annotation, species detected in both ion modes were consolidated using the preferred mode for each lipid subclass. The resulting cellular lipidome encompassed subclasses of sphingolipids, phospholipids, glycerolipids, sterol lipids, and oxidized lipids (Supplementary Table 1). Lipid subclass assignments were based on the LIPID MAPS classification system and used for lipidome profiling and lipid set enrichment analysis (LSEA).

**Fig. 1:**
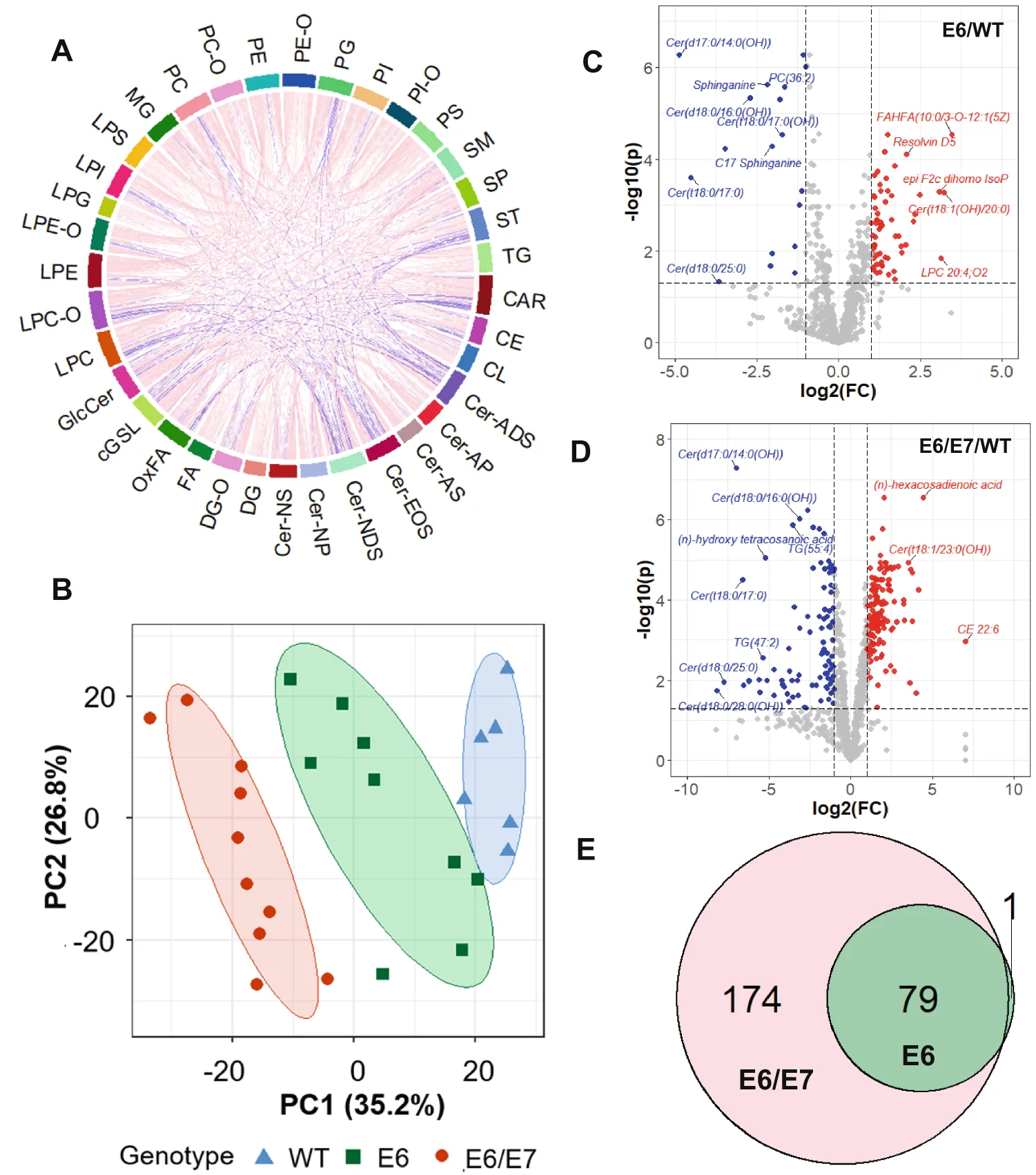
Lipidomics profiling and analysis of mouse ear skin tissue. **A** Chord diagram showing correlation relationship of lipid subclasses by Pearson’s correlation coefficient. **B** Principal component analysis (PCA) of the lipidomics dataset. The Euclidean distance of the lipid abundance among WT (n = 6), HPV E6 (n = 9) and HPV E6/E7 (n = 10) was used for sample clustering. Ellipsoids around each group indicate the distribution and spread of the samples within the sample group. **C** Volcano plots showing the distinct lipid species differentially expressed between different groups, i.e. WT (n = 6) vs E6 (n = 9), in the lipidomics data set. **D** Volcano plots showing the distinct lipids differentially expressed between different groups, i.e. WT (n = 6) vs E6/E7 (n = 10), in the lipidomics data set. Statistically significant differentially lipid species (Log2|fold change|≥ 1, FDR ≤ 0.05) are highlighted in red or blue. FC, fold change. **E** Venn diagram of E6/WT and E6/E7/WT in significantly differentiated lipid features

In *K14E6* (E6) and *K14E6/E7* (E6/E7) transgenic vs. control *FVB/N* (WT) cutaneous tissue, lysophosphatidylcholine (LPC), phosphatidylcholine (PC), and oxidized fatty acids (OxFA) were enriched, whereas most triglycerides (TG) and ceramides (Cer) were reduced as an overall trend (Supplementary Fig. 1a). Most lipid subclasses exhibited positive correlations with each other, except for various ceramide subclasses, e.g., Cer-NDS, Cer-EOS, and Cer-ADS, which were negatively correlated with phospholipids including LPC, PC, and phosphatidylglycerol (PG), acylcarnitines, and sterol lipids (Fig. 1a). Positive and negative correlations are highlighted in distinct colors, i.e. pink and blue respectively, and magnitude of correlation is represented by the depth of color. Principal component analysis (PCA) revealed distinct lipidomic profiles between HPV-positive and wild-type tissues (Fig. 1b). Lipid subclass enrichment was assessed using LSEA for comparisons between E6 vs. WT and E6/E7 vs. WT. The OxFA subclass was significantly enriched in cutaneous tissue of both E6 and E6/E7 mice compared to WT, as indicated by positive log odds ratio (LOR) values (Supplementary Table 3). Conversely, neutral glycerolipids and sphingolipid subclasses, including TG, Cer-ADS, Cer-NDS, and sphingomyelin (SM), were significantly underrepresented in both E6 and E6/E7 skin tissues. Notably, glucosylceramide (GlcCer) and cardiolipin (CL) subclasses were significantly downregulated in E6/E7 mice compared to WT. LPC and PC subclasses were upregulated in E6/E7 samples (Table 1).

**Table 1:** Epithelial and skin tissue samples from HPV oncogenic mouse model

Tissue	Genotype	Gender	N
FRT	WT	F	6
	E6	F	6
	E6/E7	F	6
Ear/skin	WT	F	6
	E6	F/M (6/3)	9
	E6/E7	F/M (6/4)	10

To identify specific lipid species altered by HPV oncoproteins, we performed differential analyses for E6 vs. WT and E6/E7 vs. WT. A total of 80 and 253 lipid species, respectively, were significantly altered (FDR ≤ 0.05, fold change > 2) in these comparisons, respectively (Fig. 1c–d). Essentially all newly identified E6-regulated lipids (79 out 80, Fig. 1e) were also observed in E6/E7 cutaneous tissue, indicating the changes are likely regulated by E6 individually and in the context of E7. All the significantly differentiated lipid species including shared and unique dysregulated lipids for E6 and E6/E7 are summarized in the Supplementary Data Table 1–10. The inferred E7-specific signatures based on volcano plot, including enrichment of cholesterol esters, decreased cardiolipins, glycosphingolipids including glucosylceramides and gangliosides, and sphingomyelins, relative to E6 in skin samples, have also shown in the direct comparisons of E6/E7 vs E6 (Supplementary Fig. 2a, Supplemental Data Table 11). In line with the LSEA results, lipid species identified by volcano plot include decreased cardiolipin, glucosylceramides, and sphingomyelin, but increased acylcarnitine, phosphatidylglycerol, and lysophosphatidylcholines in the samples expressing HPV E6/E7. Among lipid species of interest, GlcCer(d18:2/20:0) was identified via tandem MS and found to be significantly downregulated in cutaneous tissues of both oncoprotein-positive groups compared to WT, suggesting potential relevance for further study (Supplementary Fig. 3).

### Lipidomics profiling of mucosal tissues from HPV16 oncogene expressing mice

**Fig. 2:**
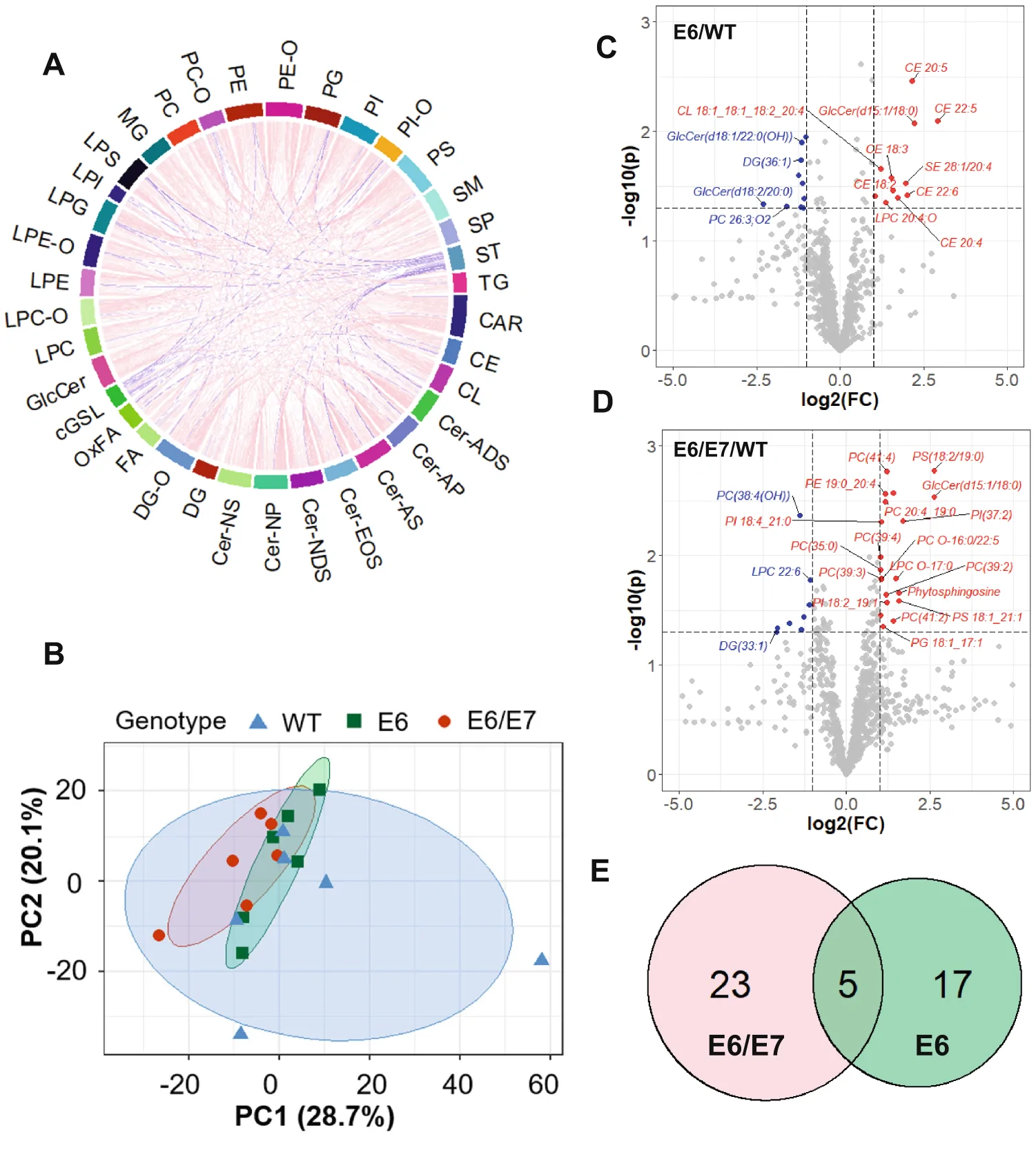
Lipidomics profiling and analysis of mouse FRT tissue. **A** Chord diagram showing correlation relationship of lipid subclasses by Pearson’s correlation coefficient. **B** Principal component analysis (PCA) of the lipidomics dataset. The Euclidean distance of the lipid abundance among WT (n = 6), HPV E6 (n = 6) and HPV E6/E7 (n = 6) was used for sample clustering. Ellipsoids around each group indicate the distribution and spread of the samples within the sample group. **C** Volcano plots showing the distinct lipid species differentially expressed between different groups, i.e. WT (n = 6) vs E6 (n = 6), in the lipidomics data set. **D** Volcano plots showing the distinct lipids differentially expressed between different groups, i.e. WT (n = 6) vs E6/E7 (n = 6), in the lipidomics data set. Statistically significant differentially lipid species (Log2|fold change|≥ 1, *P* ≤ 0.05) are highlighted in red or blue. FC, fold change. P-values shown are unadjusted (no FDR correction). **E** Venn diagram of E6/WT and E6/E7/WT in significantly differentiated lipid features

In mucosal epithelia of FRT, lipid composition analysis revealed fewer enriched lipid subclasses when compared to cutaneous tissue. The correlation chord diagram shows that most lipid subclasses were positively correlated (links shown in pink), with the exception of complex glycosphingolipids and sterol lipids, which exhibited negative correlations (links shown in blue) with phospholipids and ceramide subclasses (Fig. 2a). Among FRT samples, global multivariate clustering was not observed in the PCA score plot between groups, with WT and HPV-expressing samples (E6 and E6/E7) overlapping (Fig. 2b). LSEA results for FRT tissues revealed both similarities and differences compared to ear skin (Supplementary Table 4). Notable changes included a significant decrease (negative LOR) in neutral glycerolipids including TG and DG, but an increase in cholesterol ester (CE) in E6 and E6/E7 positive mice FRT tissues. Ether-linked LPC and PC subclasses were enriched in E6 and E6/E7 samples compared to WT. Additionally, Cer-AS was significantly underrepresented in E6/E7 samples. Volcano plots demonstrated that a total of 21 lipid species (11 upregulated, 10 downregulated) and 28 lipid species (20 upregulated, 8 downregulated) were significantly altered (unadjusted *P* ≤ 0.05, fold change > 2) in E6 vs. WT and E6/E7 vs. WT comparisons, respectively (Fig. 2c, d). To confirm the E7-induced lipid species assignment, a volcano plot of E6/E7 vs E6 was also constructed (Supplementary Fig. 2b). The inferred E7-specific signatures, including upregulation of phospholipid species in FRT samples, relative to oncoprotein E6, were also observed in the direct comparisons of E6 and E6/E7 (Supplementary Data Table 12). Notably, GlcCer(d18:2/20:0) was also downregulated in both E6 and E6/E7 FRT tissues even if not reaching statistical significance (*P* < 0.10) (Supplementary Fig. 3b).

### Improved annotation of novel lipids by molecular networking

Initial lipid annotation was performed using Progenesis QI™ software in conjunction with an in-house bulit lipidomics library, leveraging both retention time (RT) and MS/MS fragmentation spectra for database matching. While many features were reliably annotated, some ions exhibited spectral similarity to known lipids but could not be confidently identified due to their absence in the in-house library.

**Fig. 3:**
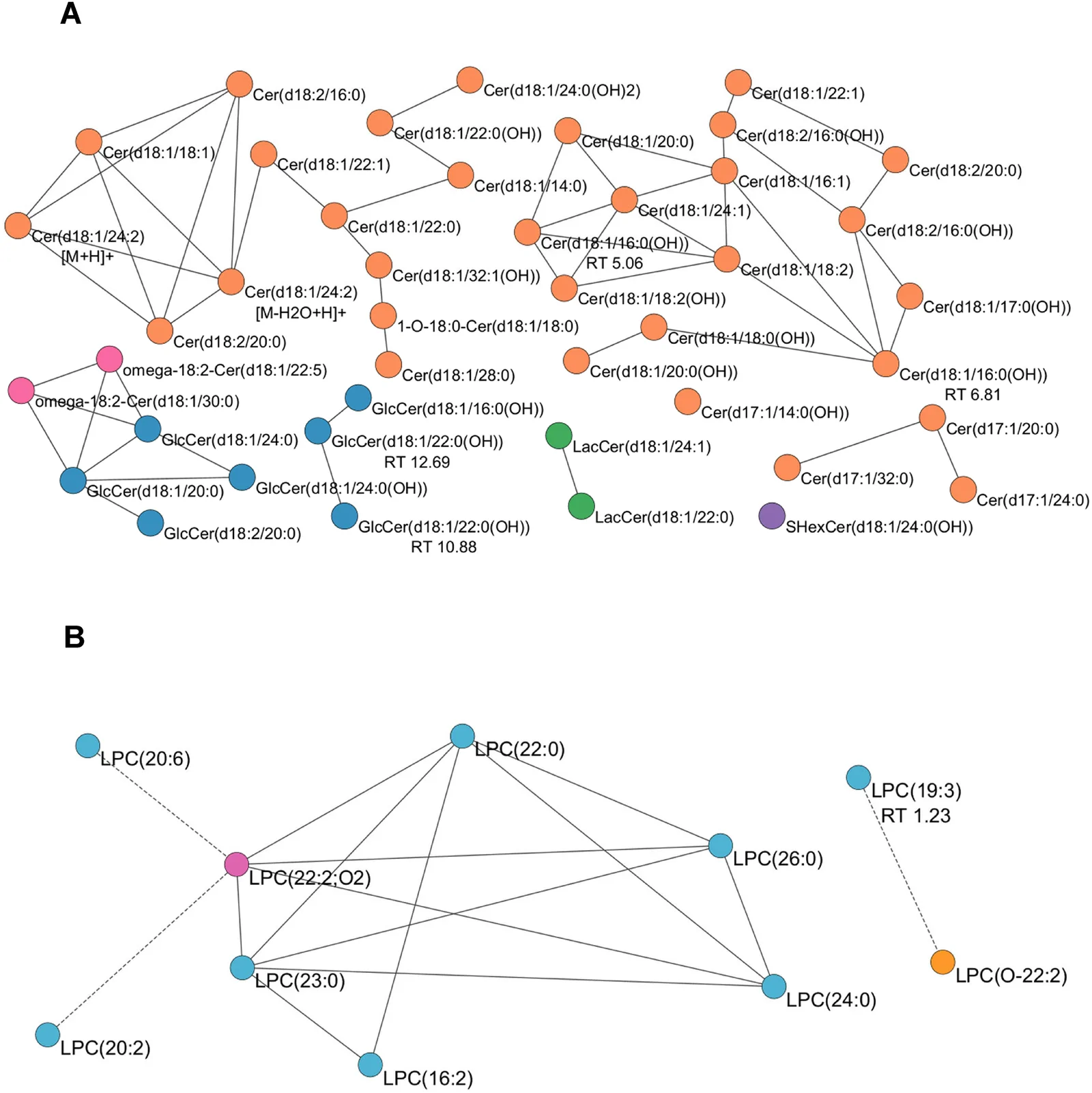

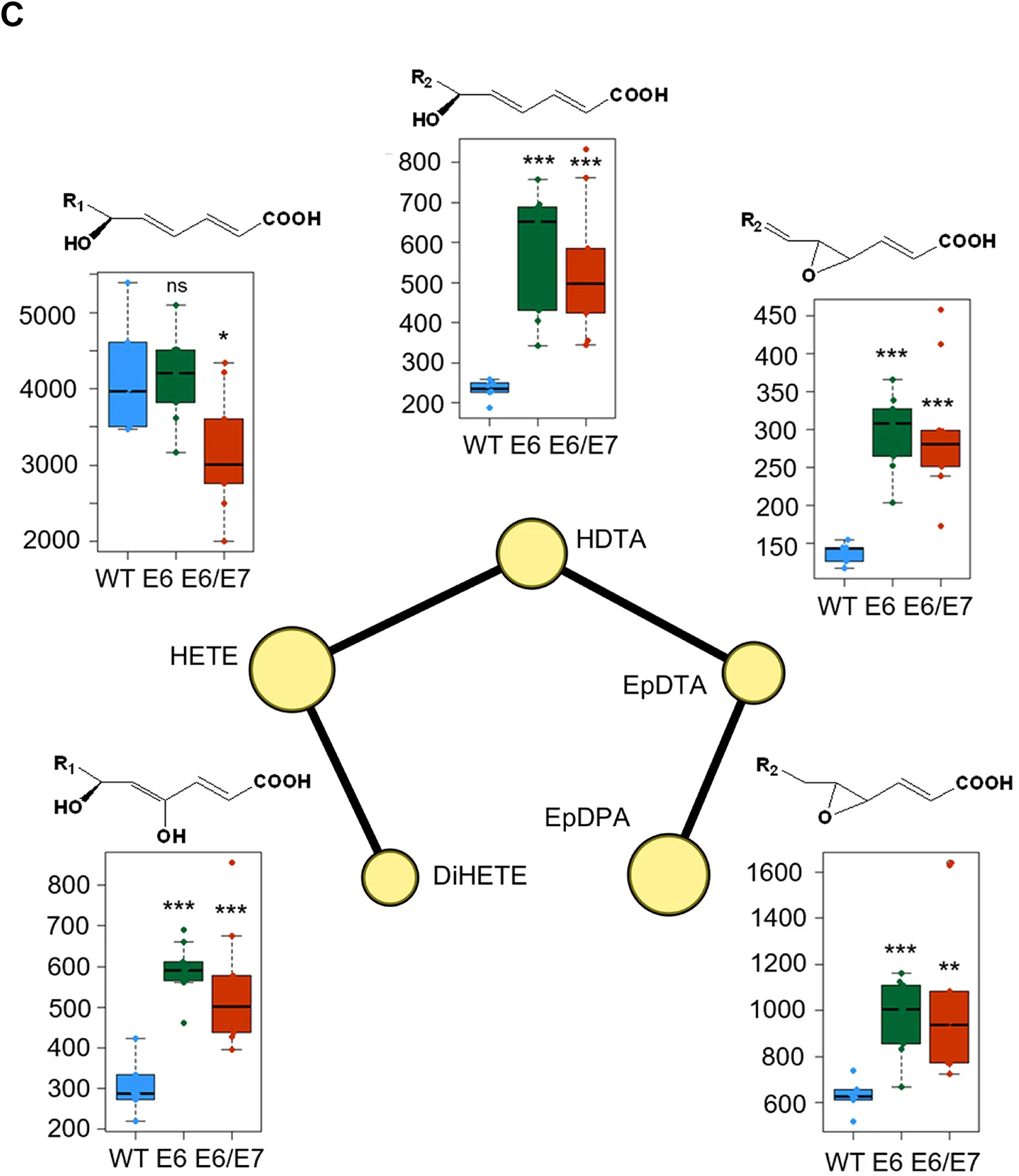
Network guided lipid species annotation by integrated Progenesis QI and GNPS analysis. **A** GNPS identified subnetwork of ceramides and hydroxylated ceramides in positive ion mode; **B** GNPS identified subnetwork of LPC and oxidized PC species from positive ion mode, dashed line represents the edge connection are indirect but less than 2 nodes in between; **C** Illustration of identified oxylipins by GNPS guided annotation, compound ion node size was proportional to ion intensity in samples. Box plot represents lipid species abundance in oncoproteins E6 (n = 9), E6/E7 (n = 10), and WT (n = 6) ear skin tissue. DiHETE, (FC = 1.9, *p* < 0.001; FC = 1.8, *p* < 0.001); HETE, (FC = 1.0, *p* = 0.99; FC = 0.8, *p* = 0.022); HDTA, (FC = 2.4, *p* < 0.001; FC = 2.3, *p* < 0.001); EpDTA, (FC = 2.1, *p* < 0.001; FC = 2.1, *p* < 0.001); EpDPA, (FC = 1.6, *p* < 0.001; FC = 1.6, *p* = 0.0046). Lipid statistical results of fold change and p-value are reported in format (E6 vs WT; E6/E7 vs WT). DiHETE: dihydroxy-eicosatetraenoic acid, HETE: hydroxy-eicosatetraenoic acid, HDTA: hydroxy-docosatetraenoic acid, EpDTA: epoxy-docosatetraenoic acid, EpDPA: epoxy-docosapentaenoic acid. Oxylipins were named generically without resolving different isomers and species

To facilitate a higher discovery, we employed molecular networking (MN) via the Global Natural Products Social Molecular Networking (GNPS) platform. Originally developed for natural products and microbial metabolites, GNPS has become increasingly valuable in metabolomics for identifying previously uncharacterized compounds (Wang et al., 2016). We hypothesized that integrating MN with our existing lipidomics pipeline would enhance both the coverage and accuracy of lipid identification. Subnetworks containing sphingolipids, phospholipids, and oxylipins were prioritized for analysis based on differential analysis (Fig. 3). To streamline the networks, we removed GNPS nodes that did not match Progenesis QI features or represented known redundant ions. Annotation was further refined by evaluating RT, lipid subclass-specific fragmentation patterns, and mass differences between connected nodes. Using classical MN, we identified a ceramide subnetwork comprising 43 nodes, all of which corresponded to Progenesis QI™ features. However, only two nodes matched entries in the GNPS spectral libraries, underscoring the limited lipid coverage in GNPS library. Despite this, MS/MS spectral similarity proved instrumental in identifying lipid molecules and we expanded the annotation of ceramides and glucosylceramides with integrating our in-house lipid library (Fig. 3a). Notably, molecular nodes clustered according to structural features such as sphingoid bases, head groups, and fatty acyl chains. For example, Cer(d18:1/24:0(OH)₂) was annotated based on a mass difference (Δm/z = 44) from Cer(d18:1/22:0(OH)), while GlcCer(d18:1/16:0(OH)) was confirmed through fragment ion similarity with GlcCer(d18:1/22:0(OH)). Additionally, a subnetwork of 11 lysophosphatidylcholine (LPC) species was generated via GNPS, supporting the annotation of this bioactive lipid subclass (Fig. 3b). Of particular interest, oxidized LPC(22:2;O₂) showed informative connections with LPC(22:0), LPC(23:0), and LPC(24:0), enabling structural inference. Both profiles of LPC(22:2;O₂) and GlcCer(d18:1/16:0(OH)) in HPV-positive skin and FRT tissues compared to WT controls are summarized in Supplementary Figs. 4, 5. Oxidized lipids are of particular interest to HPV-related research due to their roles in oxidative stress and viral pathogenesis (Foo et al., 2022; Muzammil et al., 2024).

Full-scan MS spectra of lipid hydroperoxides and hydroxides typically exhibit two dominant ions: the deprotonated ion ([M–H]⁻) and the dehydration product ([M–H–H_2_O]⁻) (Moran-Garrido et al., 2025). Collision-induced dissociation (CID) can generate characteristic fragments through cleavage adjacent to hydroxyl/hydroperoxyl groups and allylic double bonds. Molecular networking significantly enhanced the identification of oxylipins in our dataset. A dedicated subnetwork revealed profiles of hydroxyeicosatetraenoic acid (HETE), dihydroxyeicosatetraenoic acid (DiHETE), epoxydocosapentaenoic acid (EpDPA), epoxydocosatetraenoic acid (EpDTA), and hydroxydocosatetraenoic acid (HDTA) in both cutaneous (Fig. 3c) and FRT tissues (Supplementary Fig. 6) in response to HPV E6 and E6/E7 oncoprotein expression. Detailed MS/MS spectral analysis of nodes within this subnetwork improved identification of oxylipins based on diagnostic fragment ions (Supplementary Fig. 7), consistent with cleavage adjacent to epoxy or hydroxyl groups. At present, oxylipin annotations are assigned at Level 3 because isomeric ambiguity cannot be resolved by tandem mass spectrometry alone without additional analyses.

### Network LINEX^2^ analysis reveals lipid enzyme dysregulation

**Fig. 4:**
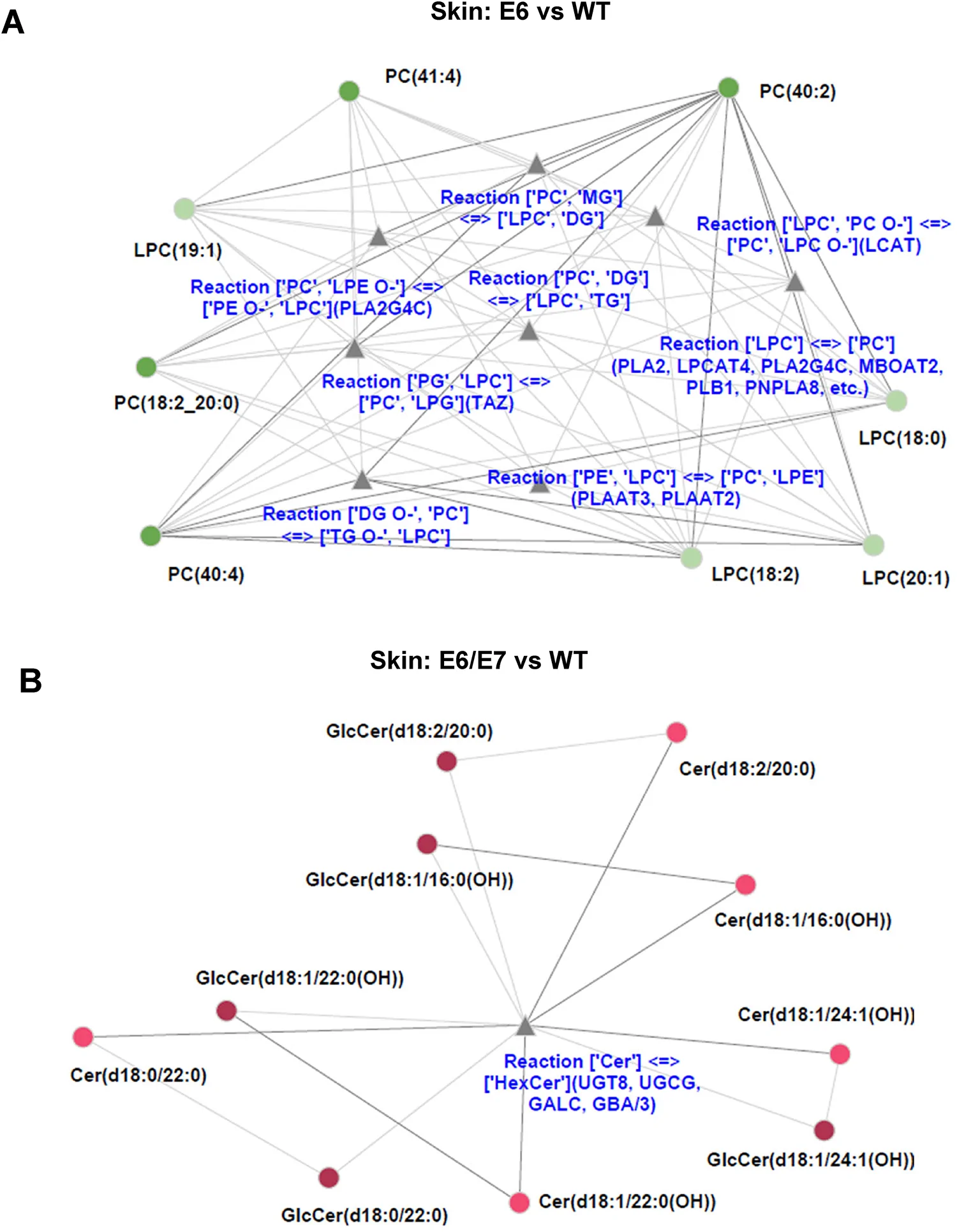

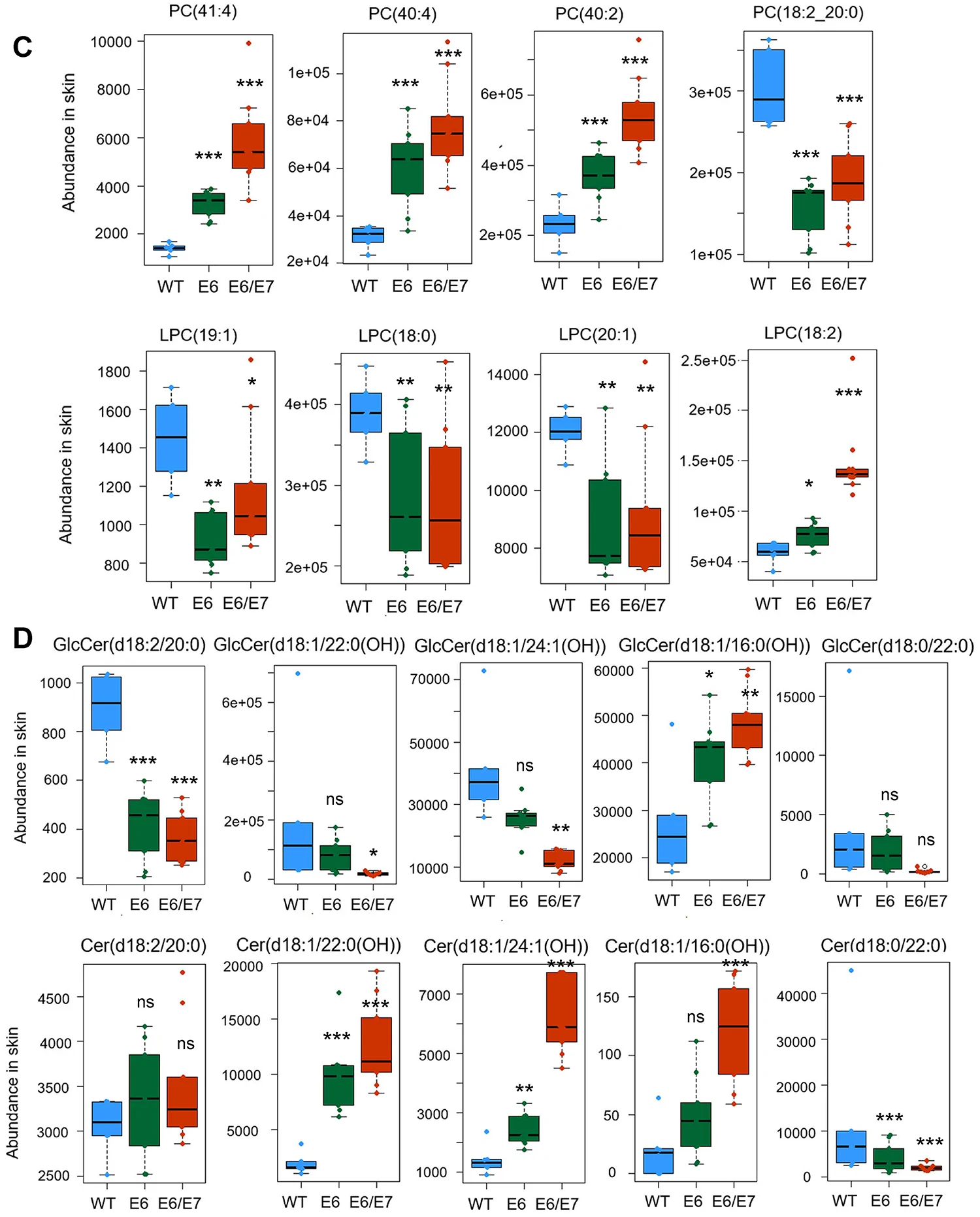

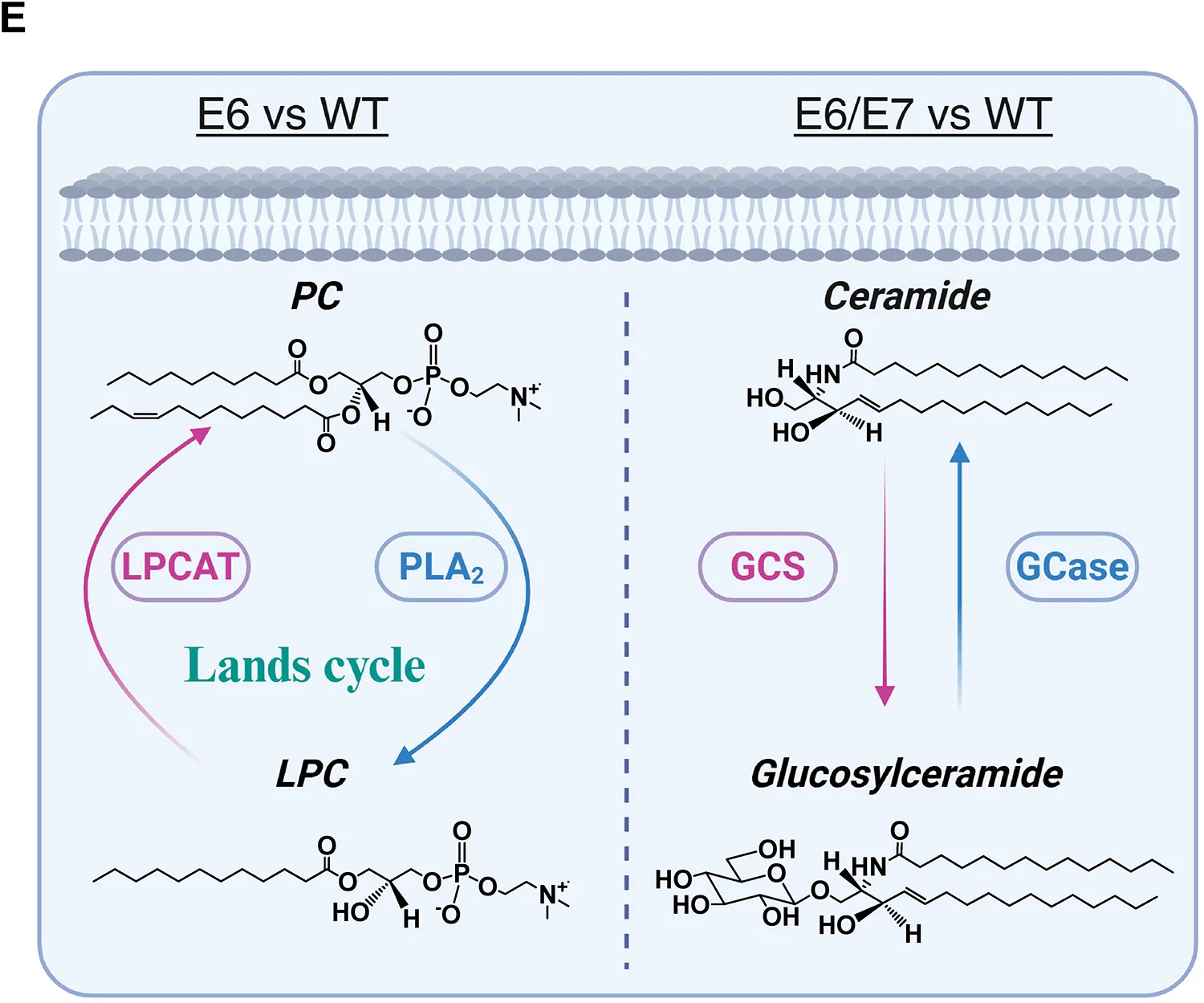
Enrichment subnetworks generated by LINEX^2^ based on global networks for ear skin tissue. **A** LINEX^2^ enrichment subnetwork for the E6 oncoprotein and WT comparison. **B** LINEX^2^ enrichment subnetwork for the E6/E7 oncoprotein and WT comparison. The subgraph with the most significant average substrate-product change are included. Data-specific lipid networks are computed based on reactions from the Rhea and Reactome databases. Spherical nodes represent lipid species, and triangular nodes represent reaction type. **C** Box plot of representative lipid species including lysophosphatidylcholine (LPC) and phosphatidylcholine (PC) from enriched subnetwork in E6 oncoprotein (n = 9) and WT (n = 6) comparisons. Lipid species are highlighted with different colors to represent the subclass. PC(41:4), (FC = 2.3, *p* < 0.001; FC = 4.1, *p* < 0.001); PC(40:4), (FC = 1.9, *p* < 0.001; FC = 2.5, *p* < 0.001); PC(40:2), (FC = 1.6, *p* < 0.001; FC = 2.3, *p* < 0.001); PC(18:2_20:0), (FC = 0.5, *p* < 0.001; FC = 0.6, *p* < 0.001); LPC(19:1), (FC = 0.6, *p* = 0.0014; FC = 0.8, *p* = 0.059); LPC(18:0), (FC = 0.8, *p* = 0.014; FC = 0.7, *p* = 0.0048); LPC(20:1), (FC = 0.7, *p* = 0.0016; FC = 0.8, *p* = 0.0046); LPC(18:2), (FC = 1.3, *p* = 0.016; FC = 2.5, *p* < 0.001). **D** Box plot of representative lipid species including glucosylceramides (GlcCer) and ceramides (Cer) from enriched subnetwork in the E6/E7 oncoprotein (n = 10) and WT (n = 6) comparison. Lipid species are highlighted with different colors to represent the subclass. GlcCer(d18:2/20:0), (FC = 0.5, *p* < 0.001; FC = 0.4, *p* < 0.001); GlcCer(d18:1/22:0(2OH)), (FC = 0.5, *p* = 0.15; FC = 0.1, *p* = 0.038); GlcCer(d18:1/24:1(OH)), (FC = 0.6, *p* = 0.069; FC = 0.3, *p* = 0.0076); GlcCer(d18:1/16:0(OH)), (FC = 1.5, *p* = 0.040; FC = 1.8, *p* = 0.0045); GlcCer(d18:0/22:0), (FC = 0.4, *p* = 0.40; FC = 0.05, *p* = 0.18); Cer(d18:2/20:0), (FC = 1.1, *p* = 0.32; FC = 1.1, *p* = 0.10); Cer(d18:1/22:0(OH)), (FC = 5.1, *p* < 0.001; FC = 6.4, *p* < 0.001); Cer(d18:1/16:0(OH)), (FC = 2.3, *p* = 0.10; FC = 6.0, *p* < 0.001); Cer(d18:1/24:1(OH)), (FC = 1.7, *p* = 0.0027; FC = 4.3, *p* < 0.001); Cer(d18:0/22:0, (FC = , *p* < 0.001; FC = , *p* < 0.001);. Lipid statistical results of fold change and p-value are reported in format (E6 vs WT; E6/E7 vs WT). P value notation in the figures follows * for p < 0.05, ** for p < 0.01, *** for p < 0.001, ns for not significant comparing to WT. **E** Schematics pathways are embedded in the subnetwork representation

**Fig. 5:**
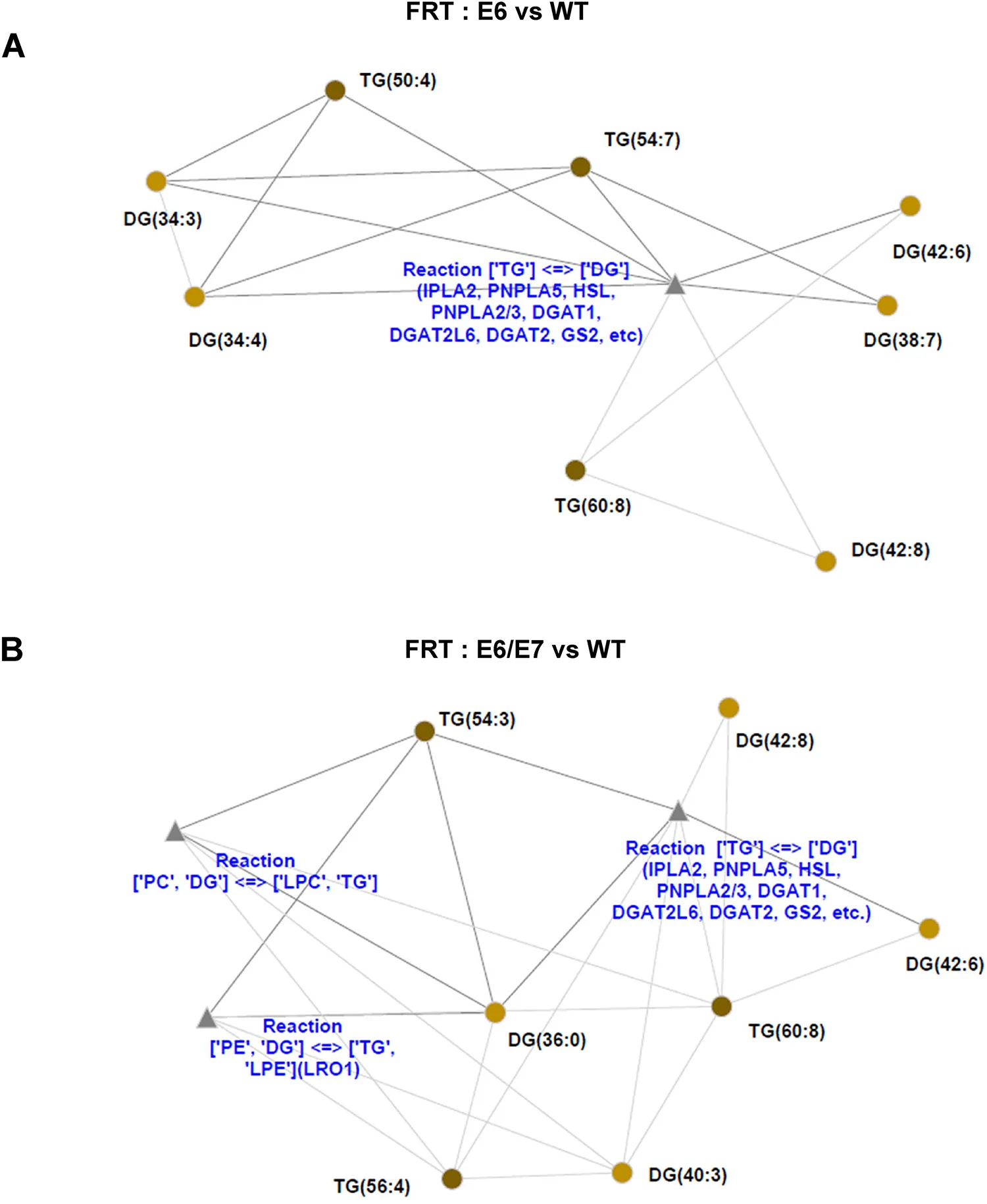

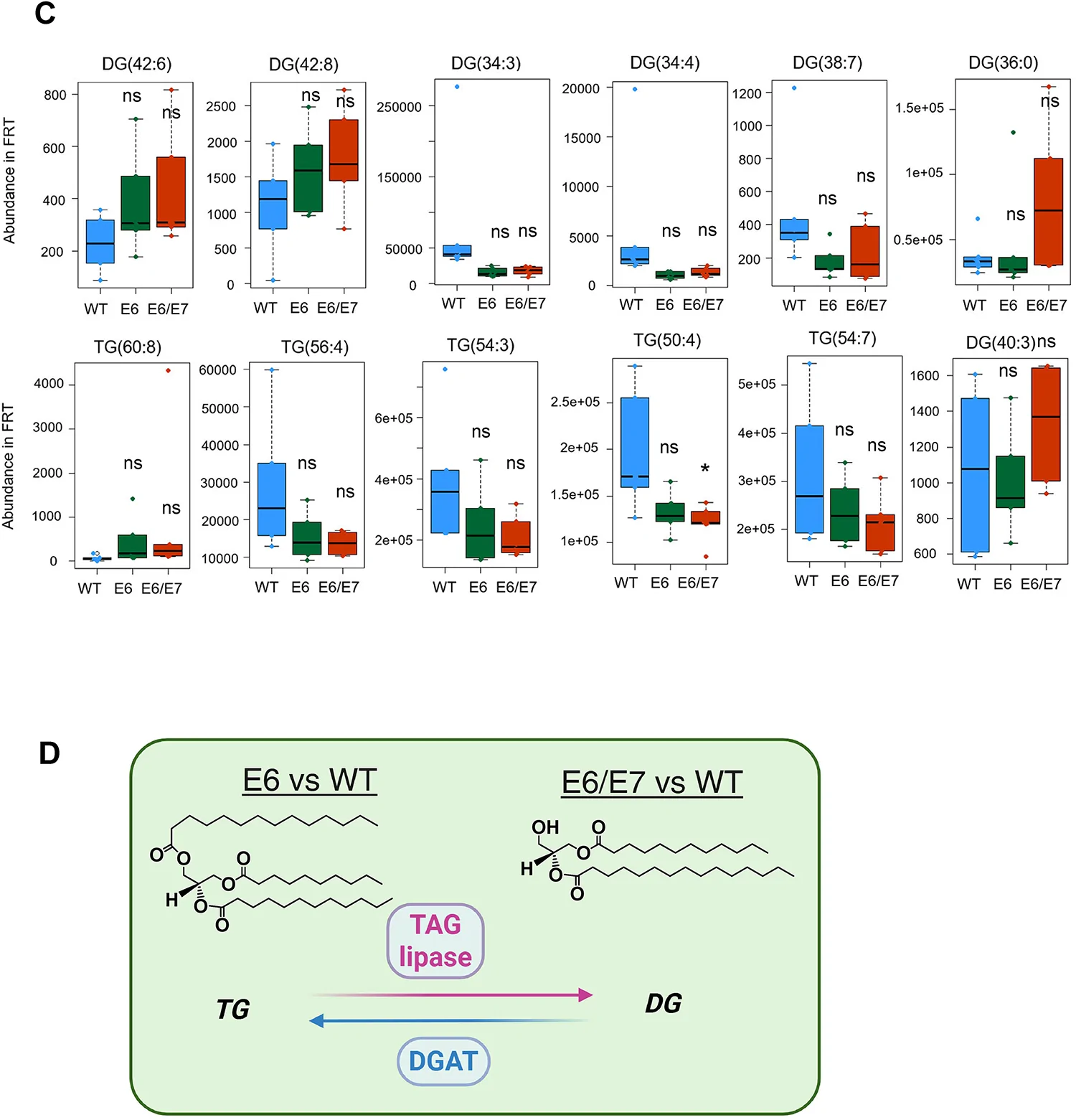
Enrichment subnetworks generated by LINEX^2^ based on global networks for FRT tissue. **A** LINEX^2^ enrichment subnetwork for the E6/E7 oncoprotein (n = 6) and WT (n = 6) comparisons in FRT tissue. **B** LINEX^2^ enrichment subnetwork for the E6 oncoprotein (n = 6) and WT (n = 6) comparisons in FRT tissue. The subgraph with the most significant average substrate-product change are included. Data-specific lipid networks are computed based on reactions from the Rhea and Reactome databases. Spherical nodes represent lipid species, and triangular nodes represent reaction type. **C** Box plot of representative lipid species including lysophosphatidylcholine (LPC) and phosphatidylcholine (PC) from enriched subnetwork. Lipid species are highlighted with different colors to represent the subclass. DG(42:6), (FC = 1.6, *p* = 0.13; FC = 1.8, *p* = 0.09); DG(42:8), (FC = 1.5, *p* = 0.20; FC = 1.6, *p* = 0.11); DG(34:3), (FC = 0.2, *p* = 0.16; FC = 0.2, *p* = 0.17); DG(34:4), (FC = 0.2, *p* = 0.18; FC = 0.2, *p* = 0.20); DG(38:7), (FC = 0.4, *p* = 0.10; FC = 0.5, *p* = 0.17); DG(36:0), (FC = 1.2, *p* = 0.70; FC = 2.2, *p* = 0.13); DG(40:3), (FC = 0.9, *p* = 0.72; FC = 1.2, *p* = 0.26); TG(60:8), (FC = 6.6, *p* = 0.15; FC = 14.1, *p* = 0.28); TG(56:4), (FC = 0.5, *p* = 0.14; FC = 0.5, *p* = 0.097); TG(54:3), (FC = 0.6, *p* = 0.17; FC = 0.5, *p* = 0.077); TG(50:4), (FC = 0.7, *p* = 0.056; FC = 0.6, *p* = 0.032); TG(54:7), (FC = , *p* = 0.93; FC = , *p* = 0.37). Lipid statistical results of fold change and p-value are reported in format (E6 vs WT; E6/E7 vs WT). **D** Schematics pathways are embedded in the subnetwork representation

We explored our lipidomics data to uncover metabolic and enzyme dysregulation. To this end, we utilized LINEX^2^, a bioinformatics tool that integrates lipid class reaction data with network enrichment algorithms to generate enriched lipid subnetworks. These subnetworks are derived from global metabolic frameworks constructed using publicly available pathway databases such as Reactome (Gillespie et al., 2022) and Rhea (Bansal et al., 2022). LINEX^2^ builds lipid species networks de novo by incorporating defined reaction types from public databases and statistical correlations. Therefore it generates dataset-specific lipid interaction networks to aid biological interpretation. Using this approach, we conducted a global network analysis comparing the HPV16 oncoprotein-expressing tissues with WT controls. This analysis enabled the identification of qualitative associations between lipid species, grounded in biochemically-defined reaction types including modification and addition/removal of headgroup and fatty acid moieties in the lipid species as illustrated in Supplementary Fig. 8 and 10. The subnetworks exhibiting the most significant average substrate–product changes were identified (Fig. 4, 5), providing mechanistic insights into potential biomarkers and metabolic pathways due to oncoprotein dysregulation of lipid metabolism in vivo.

In murine skin, the most significantly altered metabolic subnetwork in E6 vs. WT (Fig. 4a) and E6/E7 vs. WT (Fig. 4b) were lysophosphatidylcholine (LPC)-to-phosphatidylcholine (PC) conversion and glucosylceramide biosynthesis respectively, implicating the E6 protein as the viral driver. Important enzymes involved in the subnetworks include acyltransferase LCAT and phospholipase A₂ (PLA_2_) in the E6-regulated and UDP-glucose ceramide glucosyltransferase (UGCG) in the E6/E7-regulated lipidome, implicating LCAT and PLA_2_ as E6 targets and UGCG as an E7 target. Box plot of lipid species of the subnetworks show that LPC(18:2) are upregulated by E6 and E6/E7 while all other LPC species are downregulated; on the contrary, corresponding PCs are increased while only PC(18:2_20:0) decreased (Fig. 4c). This substrate-product pattern difference suggests that activities of enzymes LCAT and PLA_2_ are dysregulated with oncoprotein E6 expression. Four of five glucosylceramide species in the subnetwork decreased while the corresponding ceramide species increased, with the exception of GlcCer(d18:1/16:0(OH)) (Fig. 4d), suggesting that GlcCer synthase (UGCG) or glucocerebrosidase (GCase) are dysregulated by the E6/E7 oncoproteins. We identified enriched ontology terms in cutaneous tissue lipidomic subsets by Lipid Ontology (LION) enrichment analysis, using the lipids in the subnetwork as targets in target list mode. The E6 oncoprotein exhibited ontology terms related to membrane activity and stability, such as the lipid class term “glycerophosphocholine” and “headgroup with positive charge/zwitter-ion” (Supplementary Fig. 9a). The two most significant ontology terms associated with E6/E7 oncoprotein expression include “d18:1 Sphingosine” and “Saturated fatty acid C22:0” (Supplementary Fig. 9b).

In the murine FRT, the most dysregulated E6 and E6/E7 oncoprotein-driven subnetwork was in the metabolic reactions involving diacyglycerol (DAG) to triacylglycerol (TAG) (Fig. 5). Dysregulated enzymes in the subnetworks in HPV-positive tissues include patatin-like phospholipase domain-containing proteins (PNPLA) family which catalyze TAG hydrolysis, implicating a remodeling of glycerolipid metabolism and lipid droplet dynamics and signaling by hrHPV viral proteins. Box plots of lipid species of the dysregulated subnetworks showed that TAG species decreased in general while several DAG species were upregulated in oncoprotein expressing tissues (Fig. 5c). Lipid ontology enrichment analysis revealed dysregulation of membrane "glycerolipids", “headgroup of neutral charge”, “lipid storage” and “lipid droplet” terms (Supplementary Fig. 11). Interestingly, tissues with expression of E6 and E6/E7 relate to glycerolipid metabolism, with E6 as the likely driver. Thus, lipidome properties assigned by the LION algorithm suggest regulation of membrane resident lipids and lipid-mediated signaling upon E6 expression.

## Discussion

hrHPV infection and constitutive expression of the E6 and E7 viral oncogenes is a well-established cause of multiple squamous epithelial cancer types. The E6 and E7 oncoproteins suppress cell death and promote increased and aberrant cell proliferation via degradation of the tumor suppressor proteins p53 by E6 and Rb by E7 (Chan et al., 2019; DeFilippis et al., 2003). Joint expression of HPV16 E6 and E7 is sufficient to immortalize stratified squamous epithelial cells and promotes malignant transformation in the presence of other oncogenic hits. In our E6/E7 transgenic animals, hyperplasia can be detected at multiple sites in the skin and other sites including the mouth palate, esophagus, forestomach, and female reproductive tract (Herber et al., 1996; Song et al., 2000). These mice undergo a process of progressive neoplastic disease development, usually upon treatment with a co-carcinogen Spurgeon, M.E. (2022). This model offers an ideal platform to investigate the role of HPV oncoproteins in cellular metabolic reprogramming, with a particular focus here on lipid metabolism. Untargeted lipidomics enables the simultaneous detection of hundreds to thousands of lipid species, offering a powerful tool to define HPV-driven lipidomic dysregulation. Due to the structural diversity of lipids, untargeted lipidomics data analysis remains challenging, with lipid annotation often relying heavily on manual curation (Lei et al., 2024). To address this, we integrated Progenesis QI™ for raw data preprocessing and lipid species identification by in-house database, using molecular networking (MN) approach via the GNPS platform for new lipid species discovery. By combining outputs from both tools, we found that MN significantly improved lipid annotation coverage and accuracy.

Notably, this network-based approach expanded our coverage of sphingolipids, including hydroxyceramides and glucosylceramides. For example, in-source fragmentation in positive ion mode frequently produced water-loss adducts, particularly for ceramides and glucosylceramides. We used the [M–H_2_O + H]⁺ ion as additional evidence to support the identification of hydroxyceramides when it was the predominant molecular ion and linked to a corresponding ceramide ion in the molecular network. Sphingolipid hydroxylation patterns can disrupt specific membrane domains and exert profound impact on membrane biophysical properties (Marques et al., 2018). Viruses such as influenza are known to manipulate host cell sphingolipid metabolism to their advantage (Schneider-Schaulies et al., 2015; Thomas et al., 2023). Hydroxylated ceramides and glucosylceramides, which contain additional hydroxyl groups, exhibit distinct biological functions than their non-hydroxylated counterparts. For instance, 2′-hydroxy ceramide can be pro-apoptotic and induce apoptosis at lower concentrations (Kota et al., 2014). In mammals, all six CerS isoforms can use 2-hydroxy acyl-CoA as substrate to synthesize 2-hydroxy-FA-dihydroceramide (Mizutani et al., 2008), and continue to synthesize hydroxylated GlcCers by GlcCer synthase. Further studies, however, are needed to understand the role of these hydroxylated sphingolipids in oncoprotein expressing epithelial tissues.

Oxidized lipids, including oxylipins and oxidized phospholipids (OxPLs) were detected in both cutaneous and mucosal epithelial tissues from both control and transgenic mice. Their presence provides preliminary insight into potential lipid oxidation processes associated with hrHPV oncoprotein expression but does not establish specific pathway activation-driven malignancy. Oxylipins, which are oxidation products of polyunsaturated fatty acids (PUFAs), can arise through enzymatic mechanisms involving lipoxygenases (LOX), cyclooxygenases (COX), and cytochrome P450s, as well as through non-enzymatic oxidation driven by reactive oxygen species (ROS) under conditions of oxidative stress (Brash, 1999). Oxidative stress has been proposed as a contributing factor to hrHPV-associated carcinogenesis (Cruz-Gregorio et al., 2021), and associations between oxidative stress-related genes and HPV infection-linked cervical lesions has been reported in a recent study of 308 HPV infected women with invasive cervical carcinoma and squamous intraepithelial lesions (Inacio et al., 2023). Additionally, COX-1 and COX-2 have been implicated in HPV-related gynecological malignancies (Daikoku et al., 2006; Subbaramaiah et al., 2007).

Oxylipins encompass both pro- and anti-inflammatory species, and their biological effects depend heavily on chemical structure features and tissue context. In our dataset, dihydroxy-eicosatetraenoic acid (DiHETE), an oxylipin known to participate in inflammatory signaling pathways, was elevated in cutaneous tissues expressing hrHPV oncogenes. However, the interpretations of oxylipin alterations presented here should be viewed as hypothesis‑generating rather than conclusive evidence of inflammatory pathway activation. To firmly determine the biochemical origin and functional relevance of these oxidized lipids, future studies employing targeted MS/MS workflows, structural confirmation, and complementary biochemical assays will be essential. Notwithstanding these limitations, the molecular networking-based workflow used in this study, combined with fragmentation-guided annotation, offers a promising framework for deeper lipidome characterization and may aid in identifying oxidized lipids potentially involved in HPV16-associated pathogenesis.

In lipid metabolism, each lipid species can interact with enzymes and co-factors through multiple metabolic pathways, providing a rich foundation for investigations into their biological roles and therapeutic potential. However, bioinformatic tools that contextualize lipid species within metabolic networks remain limited. To address this gap, we used the LINEX^2^ computational framework to help interpret our lipidomics dataset. LINEX^2^ applies network‑based enrichment analyses that generate inferred patterns of potential enzymatic dysregulation, providing a hypothesis‑driven view of lipid metabolic relationships. One inferred subnetwork suggested coordinated changes involving ceramide and GlcCer metabolism. GlcCer is synthesized in Golgi/ER membranes by UDP-glucose ceramide glucosyltransferase (UGCG) and plays a critical role in membrane structure and function, which are essential for virus/host interactions. Network-based analysis revealed significant dysregulation of glycosphingolipid metabolism in the skin of *K14E6* and *K14E6/E7* HPV transgenic mice. Of note, our previous work demonstrated that the complex glycosphingolipid GM3 stimulates cancer cell invasion of head and neck squamous cell carcinoma cells harboring Fanconi anemia (FA) pathway mutations (Zhao et al., 2018). Recent studies also show that glucosylceramides are dysregulated in many cancers, with frequent upregulation of ceramide glycosyltransferases (Li et al., 2021; Schomel et al., 2020; Zhang et al., 2021). The regulation and roles of these metabolic reactions in HPV-induced infection and transformation remain to be discovered. Another enriched subnetwork by LINEX^2^ analysis involves the metabolism of lysophosphatidylcholine (LPC) and phosphatidylcholine (PC) by the HPV16 E6 oncoprotein. Both LPC and PC are increased in the E6-mediated metabolic regulation of cutaneous samples. Lysophosphatidylcholine acyltransferase 1 (LPCAT1), an enzyme involved in PC metabolism, has been implicated as an oncogene in cervical cancer (Gao et al., 2022). LPCAT1 regulates cell proliferation, epithelial–mesenchymal transition (EMT), and tumor recurrence in vitro and in vivo. Its upregulation contributes to ferroptosis resistance by increasing membrane phospholipid saturation, thereby protecting cells from peroxidation-induced membrane damage (Li et al., 2024). In addition, our current findings suggest that both E6 and E7 promote lipid oxidation, while E6 specifically disrupts glycosphingolipid biosynthesis and E7 contributes to phosphatidylcholine remodeling in host epithelial cells. Based on the comparative analysis, putative E7‑associated lipid alterations include increased phospholipids in FRT E6/E7 samples and, in skin E6/E7 samples, increased cholesterol esters accompanied by decreased cardiolipins, glycosphingolipids (including glucosylceramides and gangliosides), and sphingomyelins. We emphasize that these distinctions are inferred and remain provisional, pending validation using an E7 oncoprotein‑only transgenic mouse model. Taken together, the newly identified HPV E6 and E7 regulated lipids, along with inferred lipidomic pathways and enzymes, highlight a set of possible biomarkers and therapeutic targets that remain largely unexplored in the context of HPV-positive cancers. Our integrative approach enhances lipid annotation, enables the discovery of novel lipid species, and provides a systems-level overview of lipid metabolic reprogramming in the context of HPV oncoprotein expression.

This study has several limitations. As a pilot analysis, the sample size is relatively small. Additionally, current network-based bioinformatics tools require further automation and integration. These challenges are compounded by differences in spectral alignment methodologies between Progenesis QI™ and GNPS, which can lead to inconsistencies in peak identification. Another limitation of our study is that we did not evaluate sex-specific differences, although skin samples include both sexes. Despite these limitations, our study demonstrates the utility of combining mass spectrometry-based lipidomics with network analysis to uncover candidate lipid biomarkers in HPV16 E6 and E6/E7 transgenic mouse models. Further investigation is now warranted to better understand the respective lipid regulation and roles of E6 and E7 in manipulating host lipid pathways in high-risk HPV-induced malignancies.

## Supplementary Information

Below is the link to the electronic supplementary material.

Supplementary file1 (PPTX 3064 kb)

Supplementary file2 (DOCX 32 kb)

Supplementary file3 (XLSX 76 kb)

## Data Availability

The metabolomics and metadata reported in this paper are available as requested. This study is available at the NIH Common Fund's National Metabolomics Data Repository (NMDR) website, the Metabolomics Workbench, https://www.metabolomicsworkbench.org where it has been assigned Study ID ST004853. The data can be accessed directly via its Project DOI: http://dx.doi.org/10.21228/M87C4C (scheduled to be released on 2026-11-07).
