# Supplementary material for: Lipid reprogramming of stratified squamous epithelium by the high-risk HPV E6 and E6/E7 oncoproteins
Source: Metabolomics. 2026 Jul 1;22(4):111. doi: 10.1007/s11306-026-02498-2 (PMC13323514; doi:10.1007/s11306-026-02498-2)
Supplement: Supplementary file 1 — Supplementary file1 (PPTX 3064 kb) [file 11306_2026_2498_MOESM1_ESM.pptx]

## Slide 1
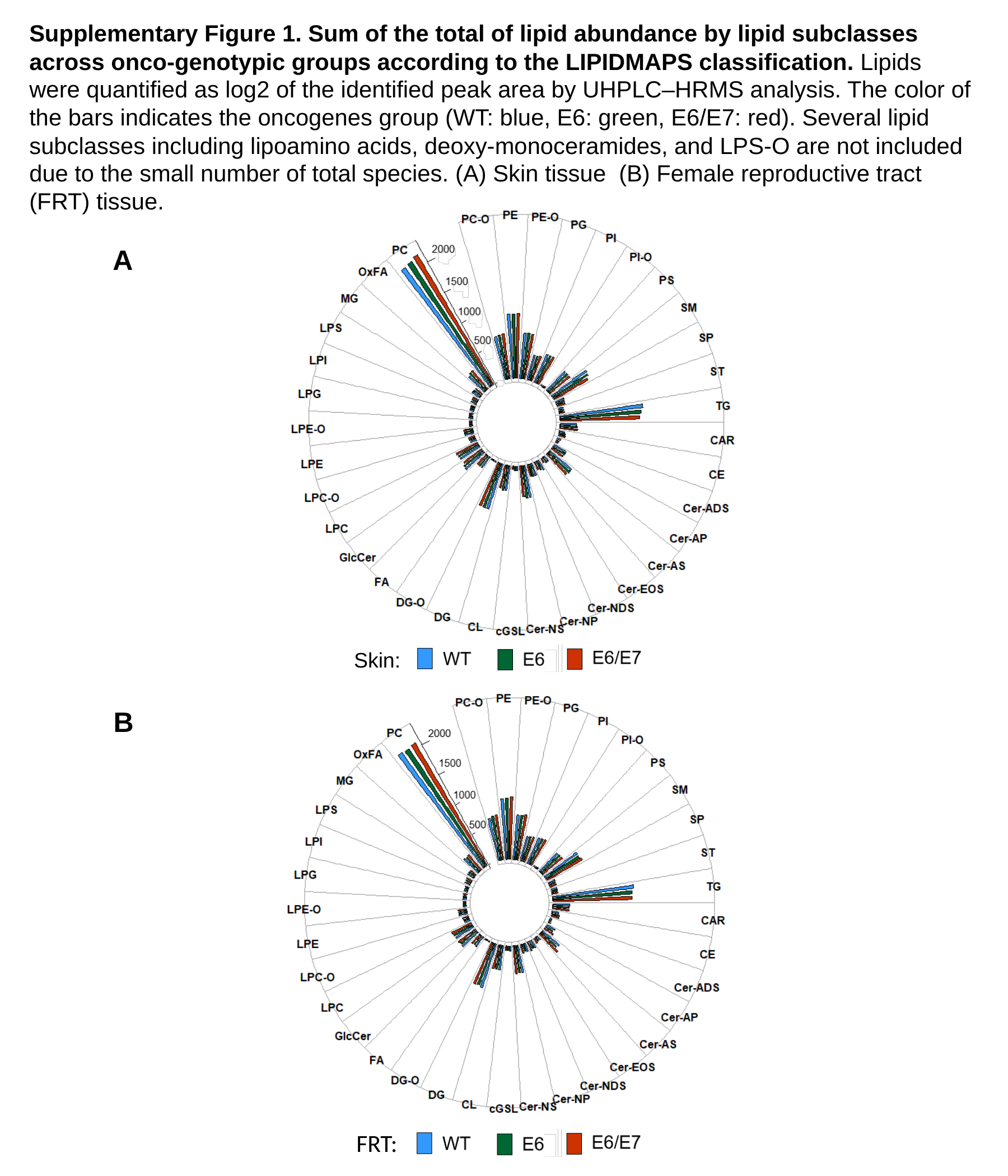

Supplementary Figure 1. Sum of the total of lipid abundance by lipid subclasses across onco-genotypic groups according to the LIPIDMAPS classification. Lipids were quantified as log2 of the identified peak area by UHPLC–HRMS analysis. The color of the bars indicates the oncogenes group (WT: blue, E6: green, E6/E7: red). Several lipid subclasses including lipoamino acids, deoxy-monoceramides, and LPS-O are not included due to the small number of total species. (A) Skin tissue (B) Female reproductive tract (FRT) tissue.
A
Skin:
B
FRT:

## Slide 2
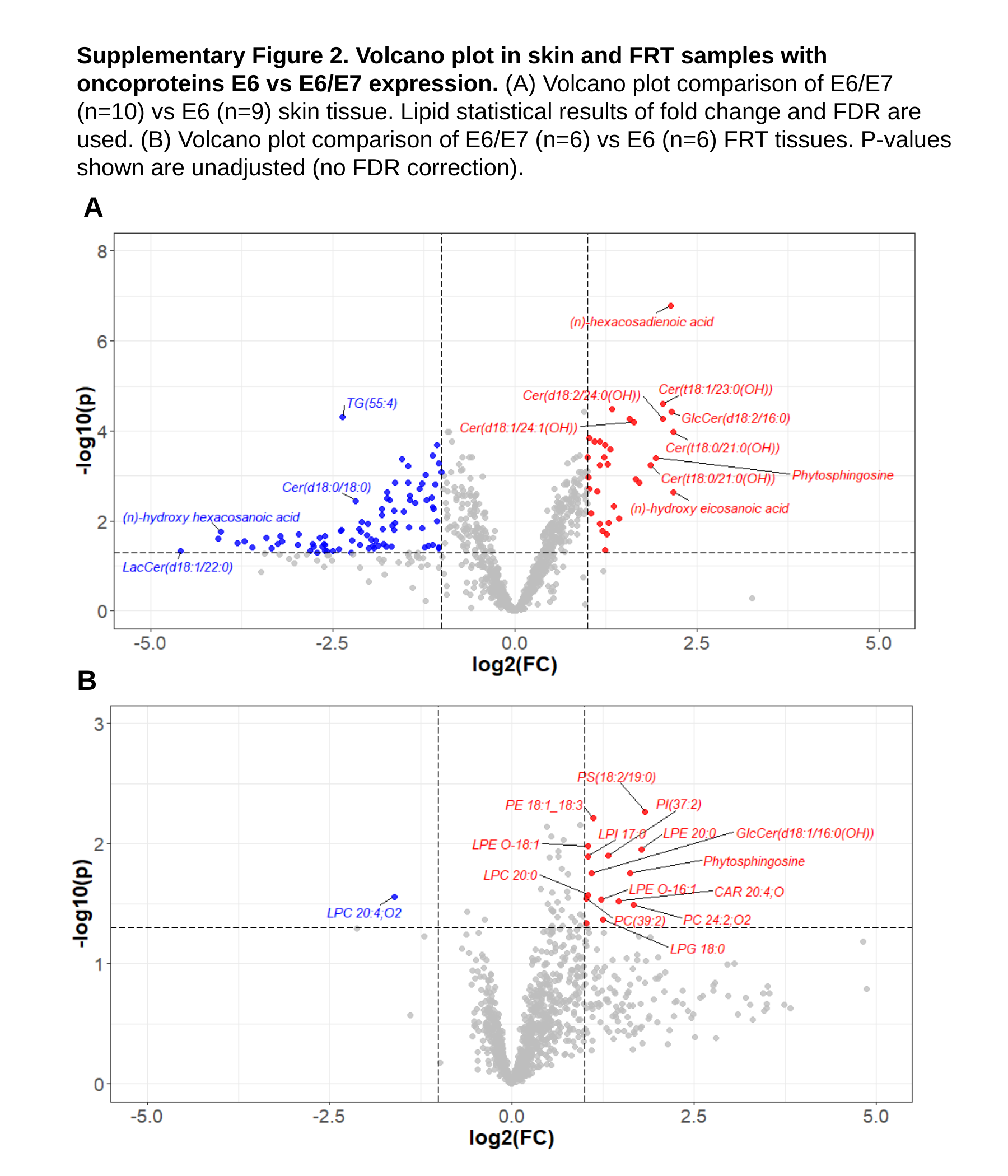

Supplementary Figure 2. Volcano plot in skin and FRT samples with oncoproteins E6 vs E6/E7 expression. (A) Volcano plot comparison of E6/E7 (n=10) vs E6 (n=9) skin tissue. Lipid statistical results of fold change and FDR are used. (B) Volcano plot comparison of E6/E7 (n=6) vs E6 (n=6) FRT tissues. P‑values shown are unadjusted (no FDR correction).
A
B

## Slide 3
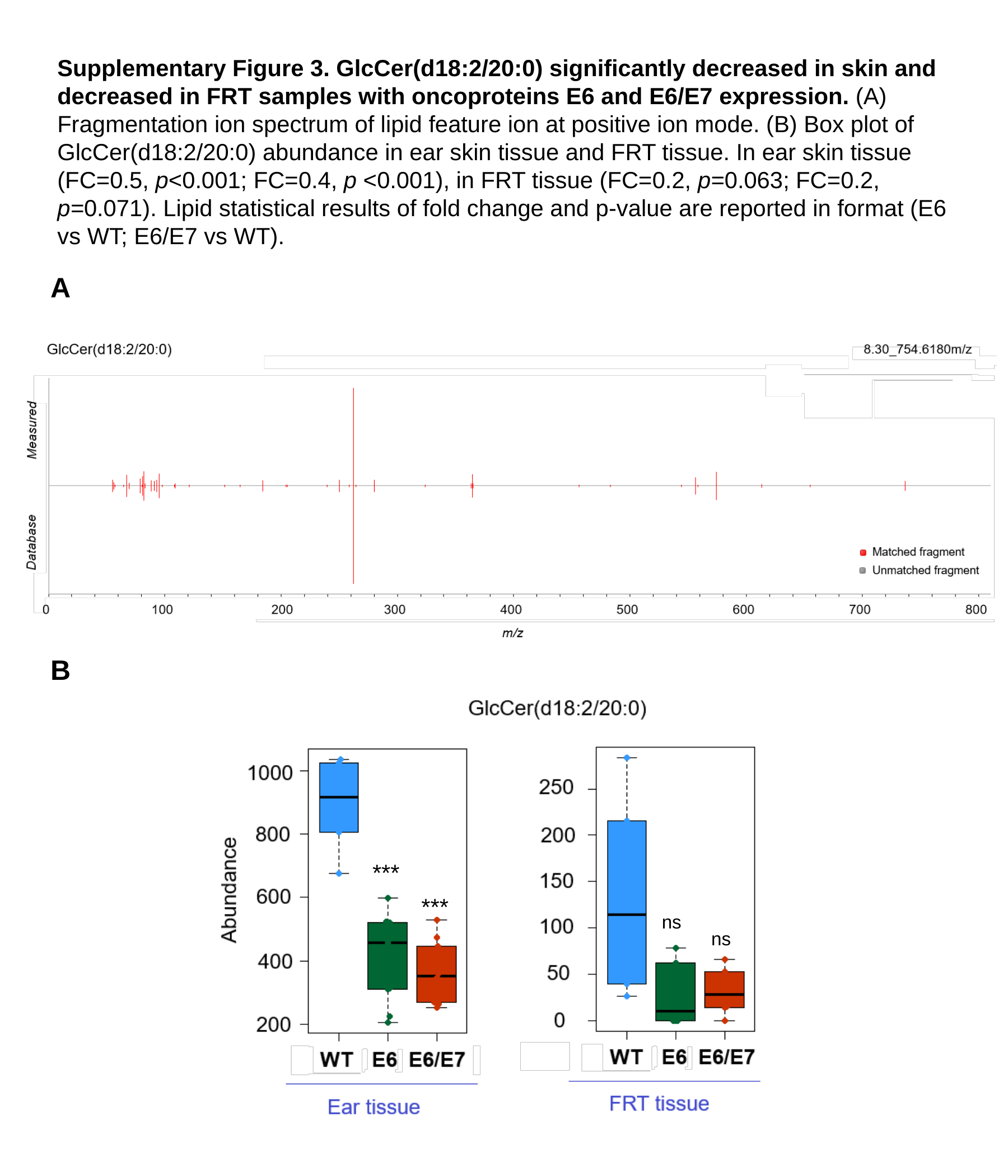

Supplementary Figure 3. GlcCer(d18:2/20:0) significantly decreased in skin and decreased in FRT samples with oncoproteins E6 and E6/E7 expression. (A) Fragmentation ion spectrum of lipid feature ion at positive ion mode. (B) Box plot of GlcCer(d18:2/20:0) abundance in ear skin tissue and FRT tissue. In ear skin tissue (FC=0.5, p<0.001; FC=0.4, p <0.001), in FRT tissue (FC=0.2, p=0.063; FC=0.2, p=0.071). Lipid statistical results of fold change and p-value are reported in format (E6 vs WT; E6/E7 vs WT).
A
B
***
***
ns
ns

## Slide 4
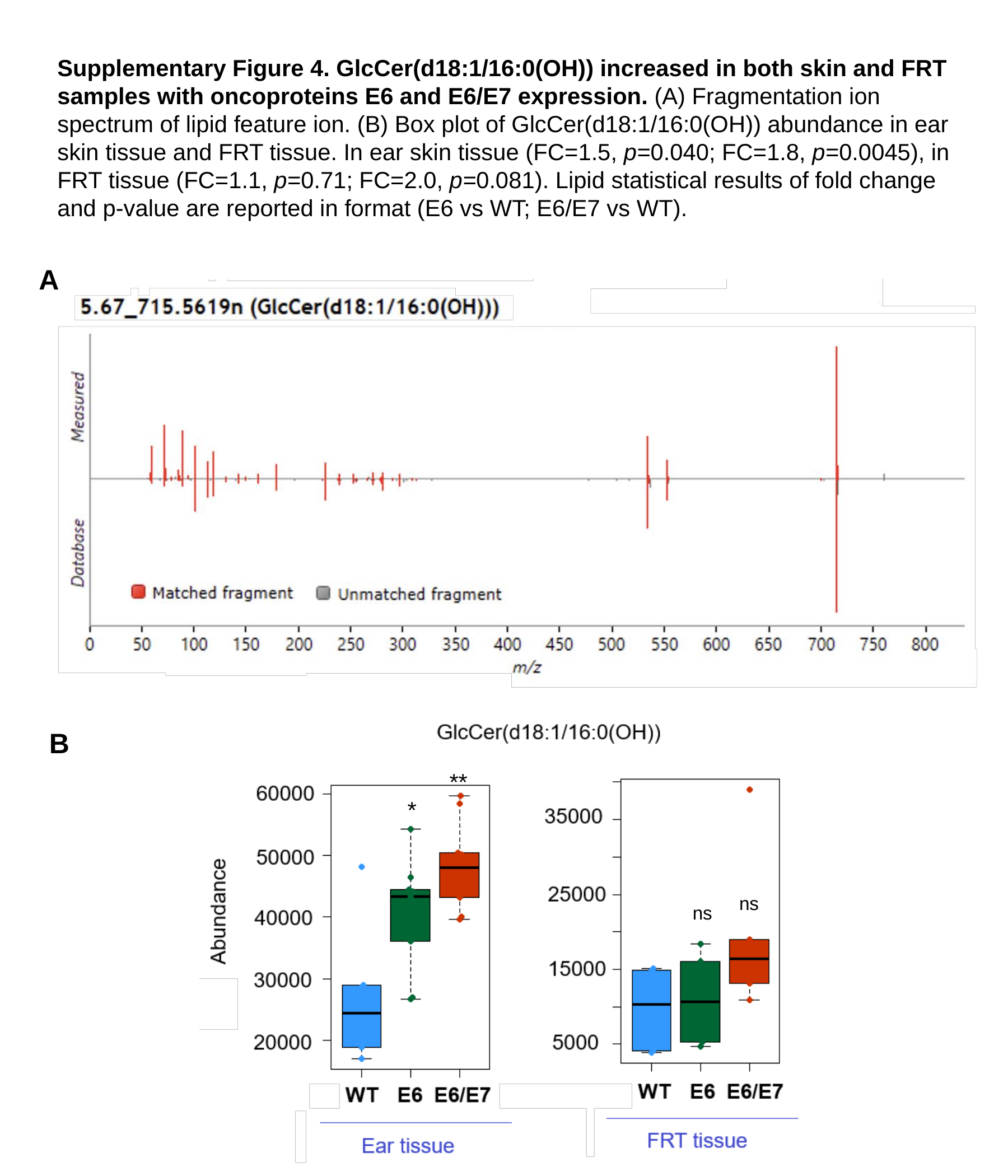

Supplementary Figure 4. GlcCer(d18:1/16:0(OH)) increased in both skin and FRT samples with oncoproteins E6 and E6/E7 expression. (A) Fragmentation ion spectrum of lipid feature ion. (B) Box plot of GlcCer(d18:1/16:0(OH)) abundance in ear skin tissue and FRT tissue. In ear skin tissue (FC=1.5, p=0.040; FC=1.8, p=0.0045), in FRT tissue (FC=1.1, p=0.71; FC=2.0, p=0.081). Lipid statistical results of fold change and p-value are reported in format (E6 vs WT; E6/E7 vs WT).
A
B
**
*
ns
ns

## Slide 5
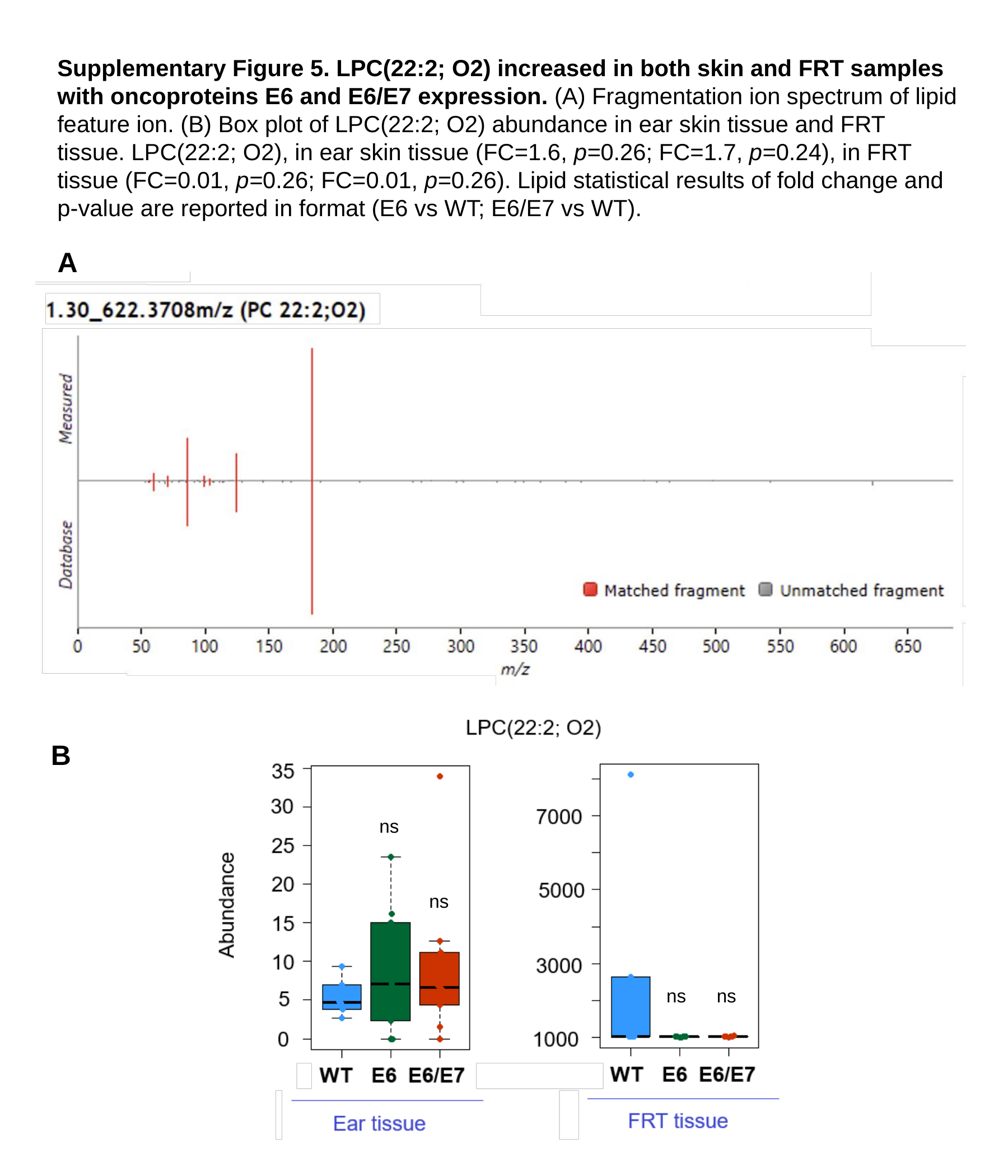

Supplementary Figure 5. LPC(22:2; O2) increased in both skin and FRT samples with oncoproteins E6 and E6/E7 expression. (A) Fragmentation ion spectrum of lipid feature ion. (B) Box plot of LPC(22:2; O2) abundance in ear skin tissue and FRT tissue. LPC(22:2; O2), in ear skin tissue (FC=1.6, p=0.26; FC=1.7, p=0.24), in FRT tissue (FC=0.01, p=0.26; FC=0.01, p=0.26). Lipid statistical results of fold change and p-value are reported in format (E6 vs WT; E6/E7 vs WT).
A
B
ns
ns
ns
ns

## Slide 6
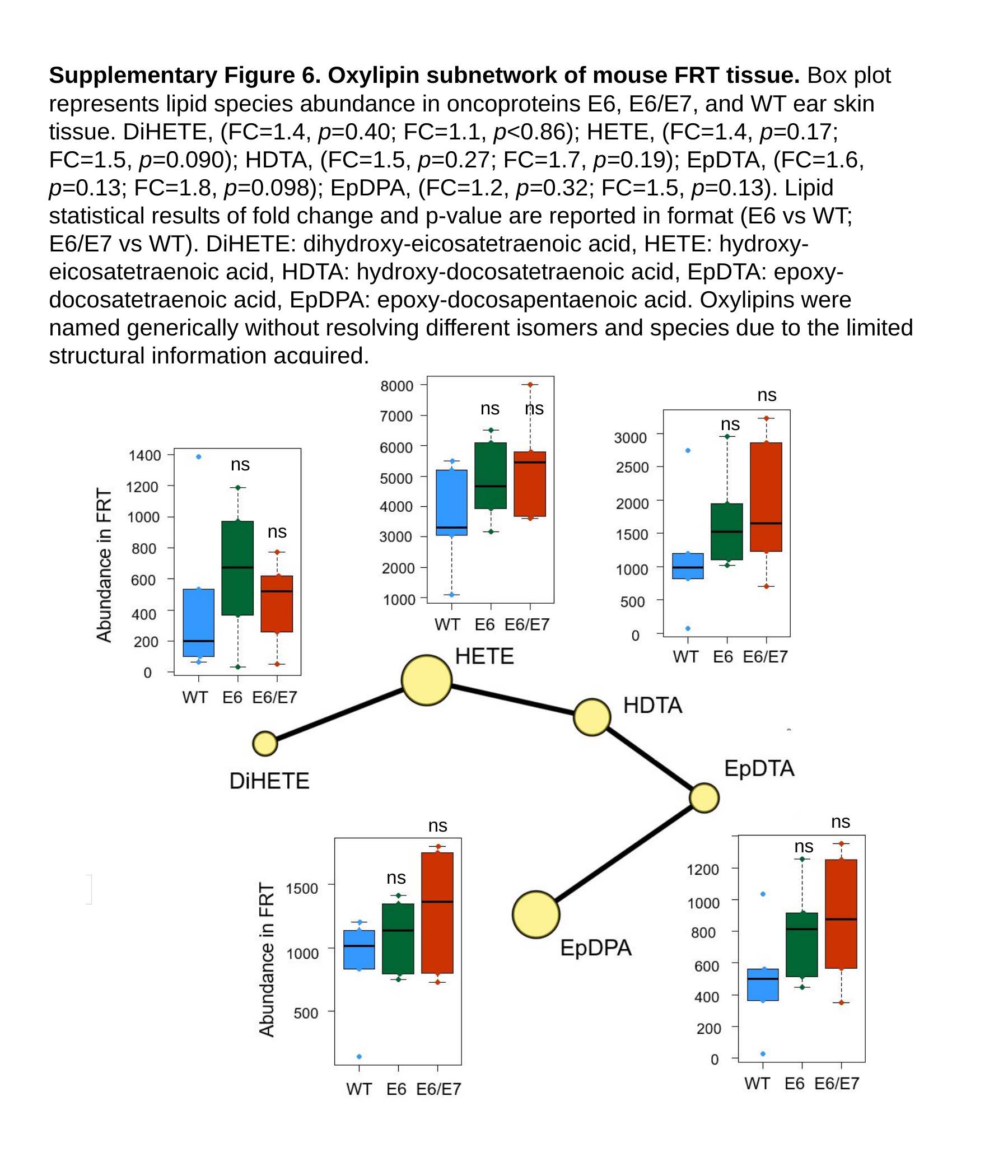

Supplementary Figure 6. Oxylipin subnetwork of mouse FRT tissue. Box plot represents lipid species abundance in oncoproteins E6, E6/E7, and WT ear skin tissue. DiHETE, (FC=1.4, p=0.40; FC=1.1, p<0.86); HETE, (FC=1.4, p=0.17; FC=1.5, p=0.090); HDTA, (FC=1.5, p=0.27; FC=1.7, p=0.19); EpDTA, (FC=1.6, p=0.13; FC=1.8, p=0.098); EpDPA, (FC=1.2, p=0.32; FC=1.5, p=0.13). Lipid statistical results of fold change and p-value are reported in format (E6 vs WT; E6/E7 vs WT). DiHETE: dihydroxy-eicosatetraenoic acid, HETE: hydroxy-eicosatetraenoic acid, HDTA: hydroxy-docosatetraenoic acid, EpDTA: epoxy-docosatetraenoic acid, EpDPA: epoxy-docosapentaenoic acid. Oxylipins were named generically without resolving different isomers and species due to the limited structural information acquired.
ns
ns
ns
ns
ns
ns
ns
ns
ns
ns

## Slide 7
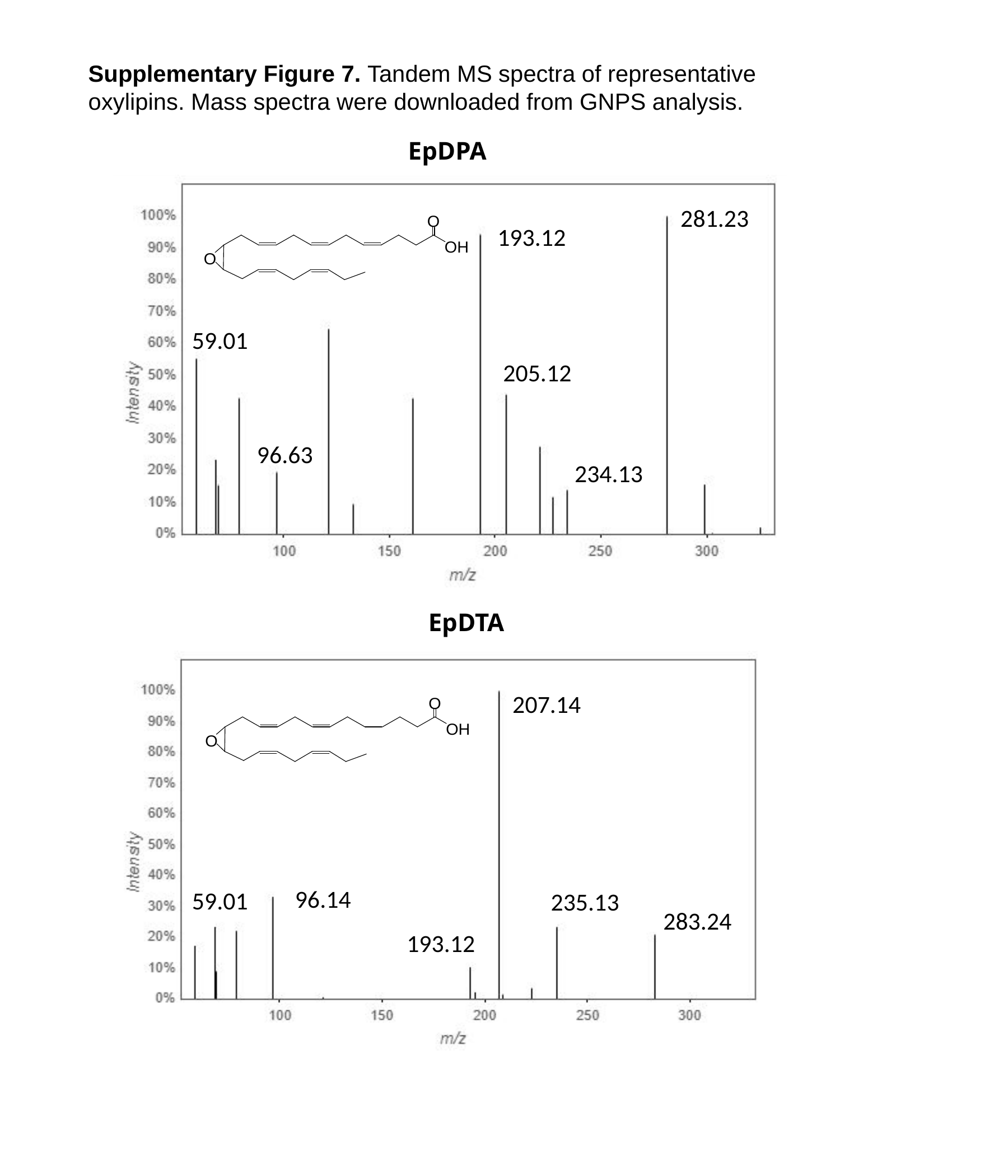

Supplementary Figure 7. Tandem MS spectra of representative oxylipins. Mass spectra were downloaded from GNPS analysis.
EpDPA
281.23
193.12
59.01
205.12
96.63
234.13
EpDTA
207.14
96.14
59.01
235.13
283.24
193.12

## Slide 8
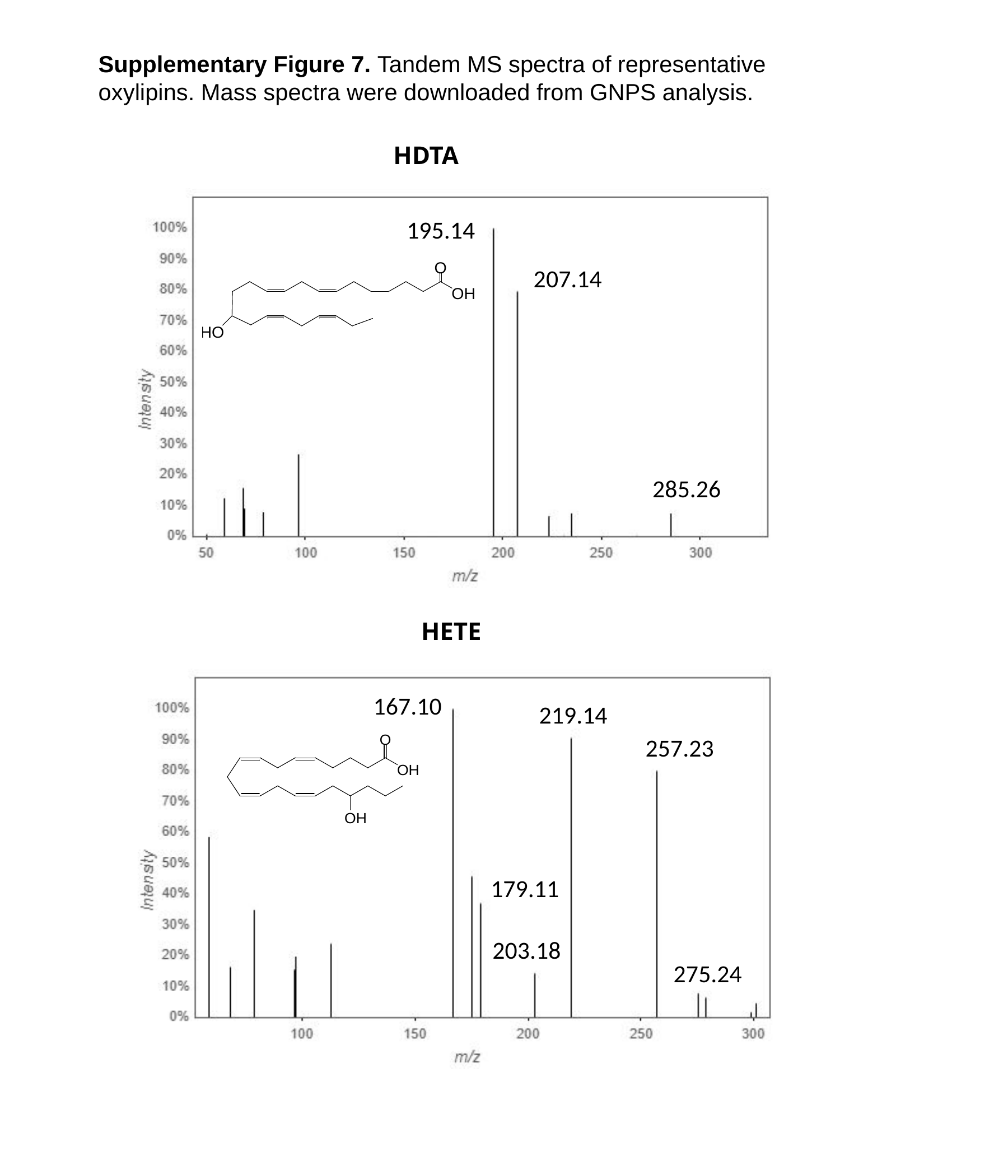

Supplementary Figure 7. Tandem MS spectra of representative oxylipins. Mass spectra were downloaded from GNPS analysis.
HDTA
195.14
207.14
285.26
HETE
167.10
219.14
257.23
179.11
203.18
275.24

## Slide 9
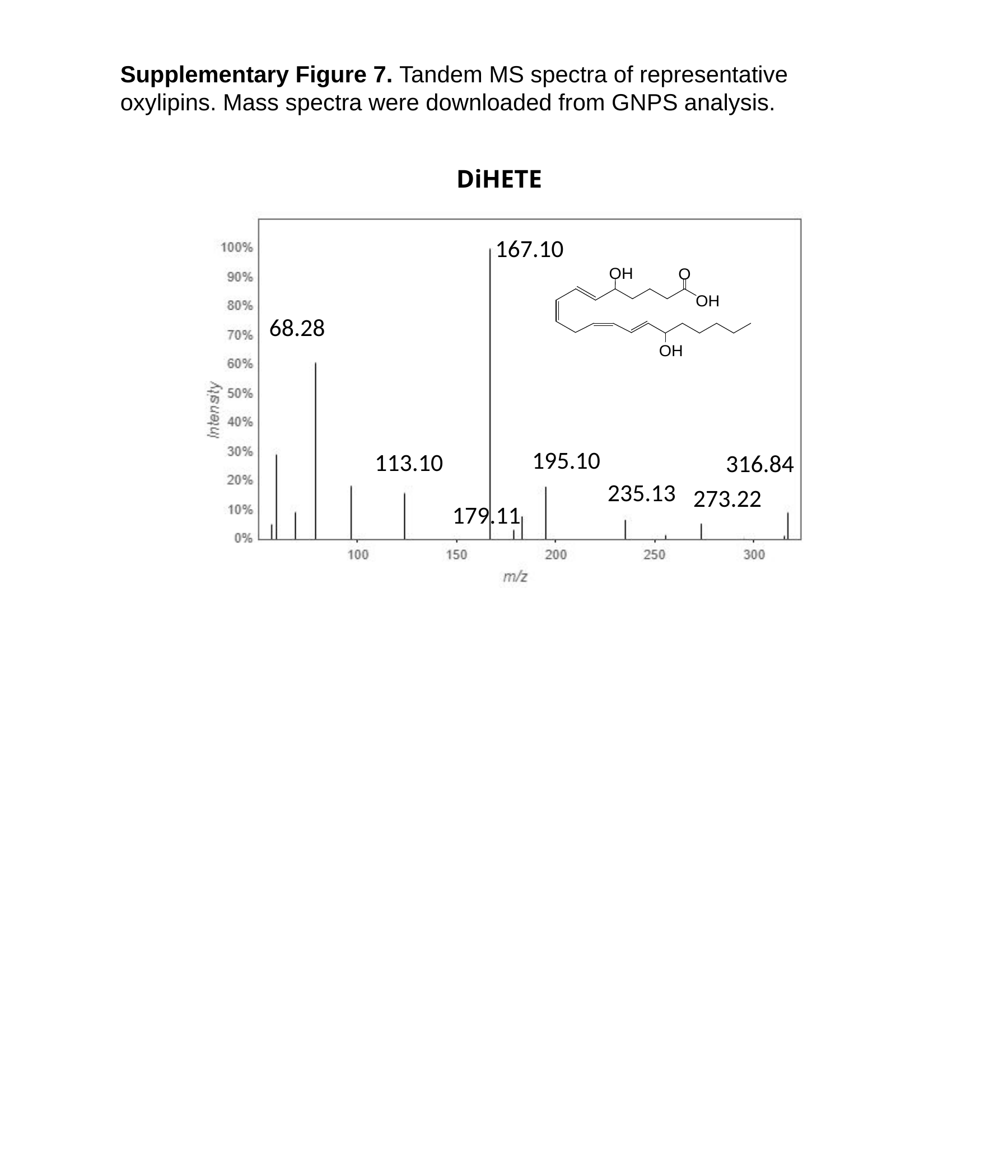

Supplementary Figure 7. Tandem MS spectra of representative oxylipins. Mass spectra were downloaded from GNPS analysis.
DiHETE
167.10
68.28
195.10
113.10
316.84
235.13
273.22
179.11

## Slide 10
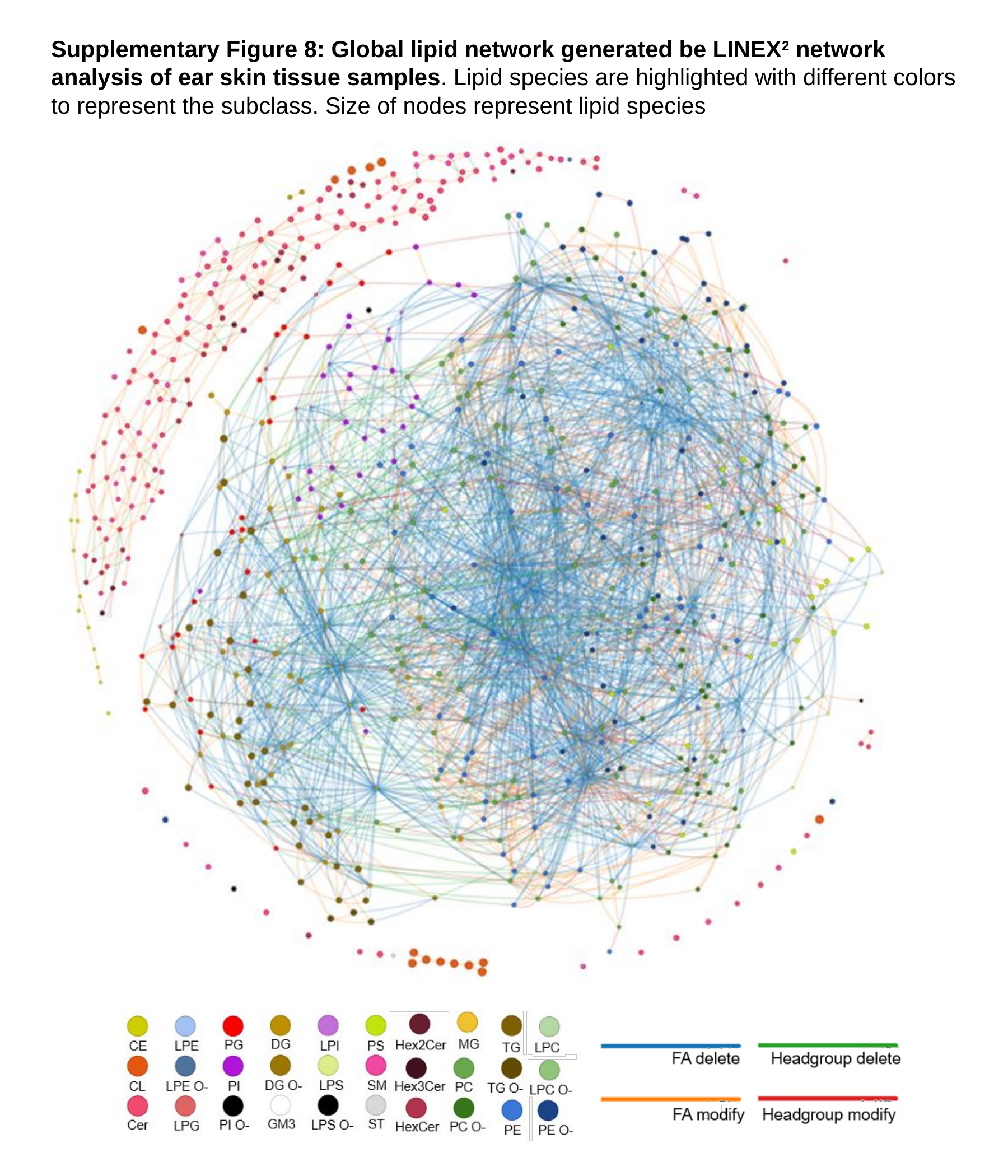

Supplementary Figure 8: Global lipid network generated be LINEX2 network analysis of ear skin tissue samples. Lipid species are highlighted with different colors to represent the subclass. Size of nodes represent lipid species

## Slide 11
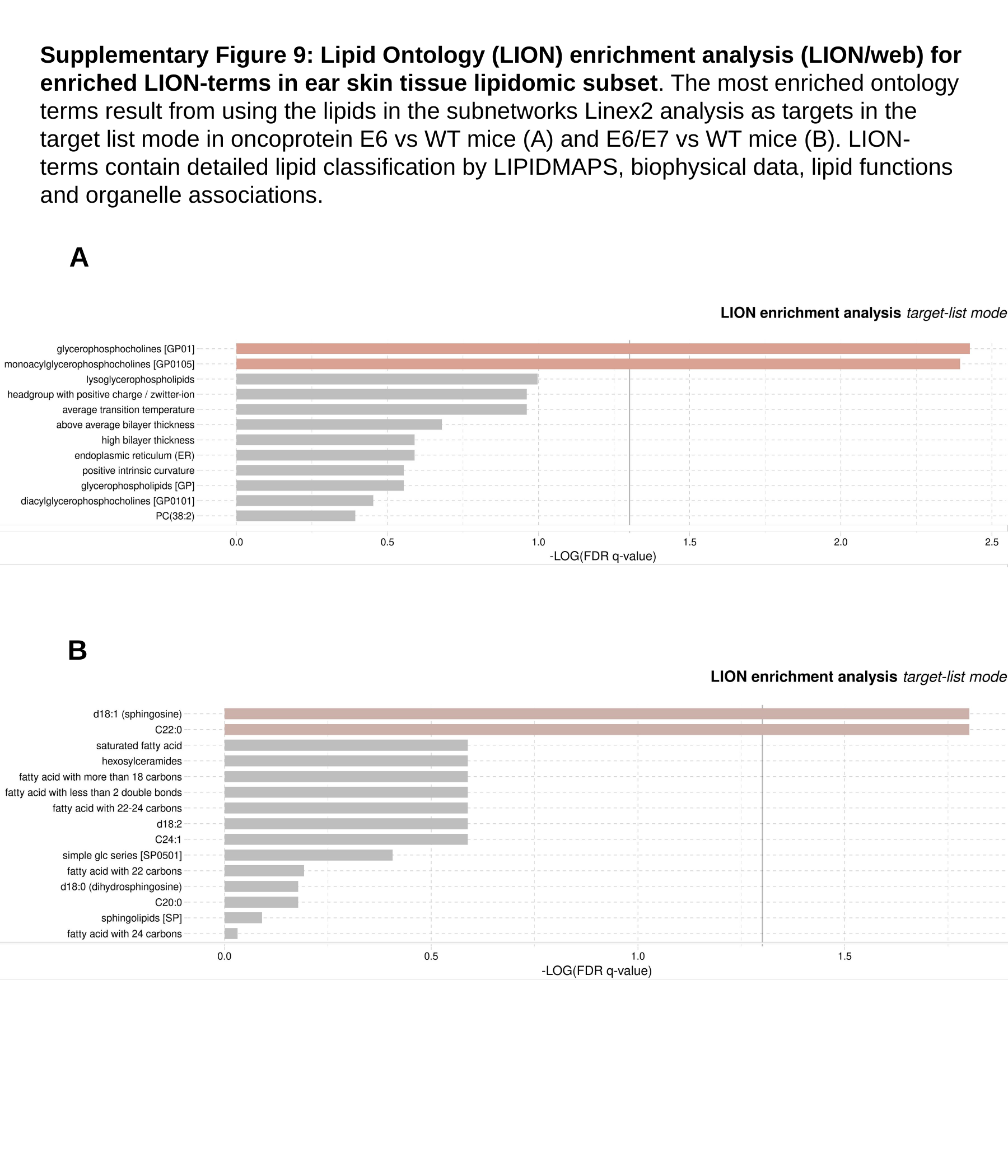

Supplementary Figure 9: Lipid Ontology (LION) enrichment analysis (LION/web) for enriched LION-terms in ear skin tissue lipidomic subset. The most enriched ontology terms result from using the lipids in the subnetworks Linex2 analysis as targets in the target list mode in oncoprotein E6 vs WT mice (A) and E6/E7 vs WT mice (B). LION-terms contain detailed lipid classification by LIPIDMAPS, biophysical data, lipid functions and organelle associations.
A
B

## Slide 12
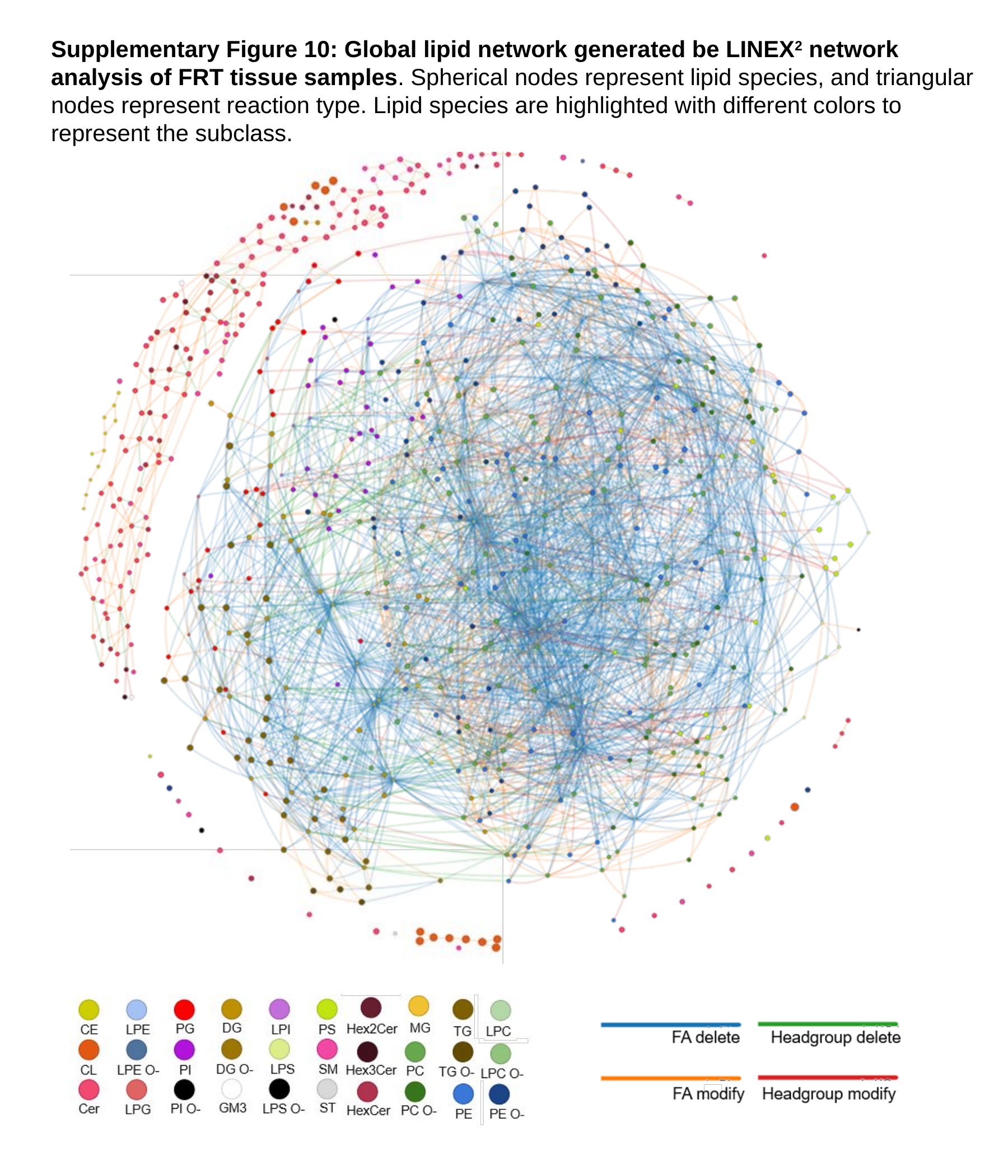

Supplementary Figure 10: Global lipid network generated be LINEX2 network analysis of FRT tissue samples. Spherical nodes represent lipid species, and triangular nodes represent reaction type. Lipid species are highlighted with different colors to represent the subclass.

## Slide 13
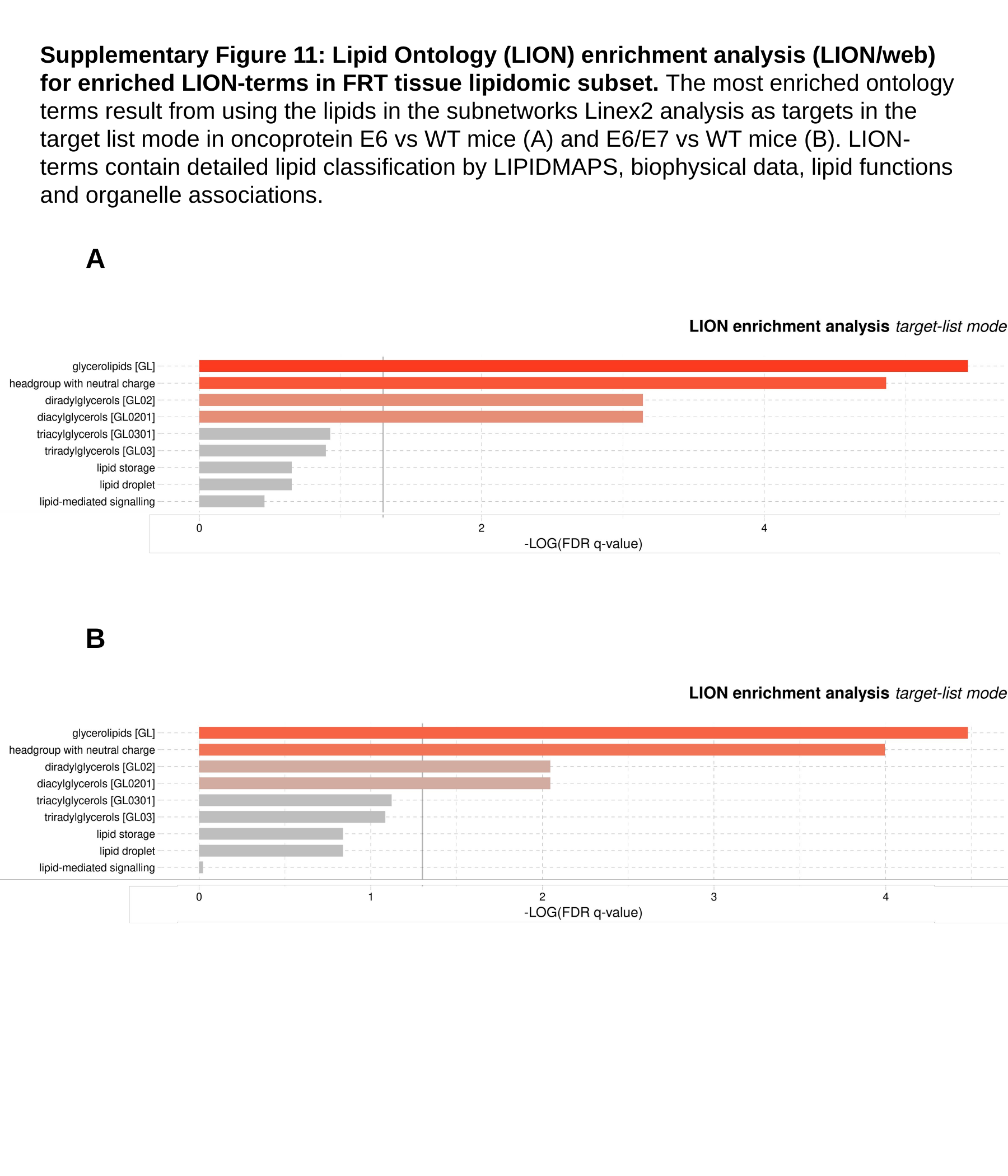

Supplementary Figure 11: Lipid Ontology (LION) enrichment analysis (LION/web) for enriched LION-terms in FRT tissue lipidomic subset. The most enriched ontology terms result from using the lipids in the subnetworks Linex2 analysis as targets in the target list mode in oncoprotein E6 vs WT mice (A) and E6/E7 vs WT mice (B). LION-terms contain detailed lipid classification by LIPIDMAPS, biophysical data, lipid functions and organelle associations.
A
B
