# Supplementary material for: Lipid reprogramming of stratified squamous epithelium by the high-risk HPV E6 and E6/E7 oncoproteins
Source: Metabolomics. 2026 Jul 1;22(4):111. doi: 10.1007/s11306-026-02498-2 (PMC13323514; doi:10.1007/s11306-026-02498-2)
Supplement: Supplementary file 2 — Supplementary file2 (DOCX 32 kb) [file 11306_2026_2498_MOESM2_ESM.docx]

**Supplementary Table 1**. Classification by levels of all lipids in samples.

| Super class | Main class | Subclass | Shorthand name | N |
| --- | --- | --- | --- | --- |
| Sphingolipids | Phosphosphingolipids | Sphingomyelin | SM | 44 |
|  | Glycosphingolipids | Glucosylceramide | GlcCer | 30 |
|  |  | Complex glycosphingolipids | cGSL | 8 |
|  | Ceramides | 1-deoxy-ceramide | 1D-Cer-S | 2 |
|  |  | Alpha-hydroxy- dihydrosphingosine | Cer-ADS | 5 |
|  |  | Alpha-hydroxy- phytosphingosine | Cer-AP | 22 |
|  |  | Alpha-hydroxy-fatty acid sphingosine | Cer-AS | 39 |
|  |  | Esterified omega-hydroxylated-acyl sphingosine | Cer-EOS | 9 |
|  |  | Non-hydroxy- dihydrosphingosine | Cer-NDS | 14 |
|  |  | Non-hydroxy- phytosphingosine | Cer-NP | 16 |
|  |  | Non-hydroxy- sphingosine | Cer-NS | 40 |
|  | Other sphingolipids | Other sphingolipid | SP | 11 |
| Fatty Acyls | Fatty ester | Fatty acyl carnitine | CAR | 24 |
|  | Fatty amide | N-acyl amino acid | FA-AA | 2 |
|  | Fatty acids | Fatty acid | FA | 23 |
|  |  | Oxidized fatty acid | OxFA | 41 |
| Glycerophospholipids | Glycerophosphocholines | Phosphatidylcholine | PC | 157 |
|  |  | Ether-linked phosphatidylcholine | PC-O | 51 |
|  |  | Lysophosphatidylcholine | LPC | 27 |
|  |  | Ether-linked lysophosphatidylcholine | LPC-O | 9 |
|  | Glycerophosphoethanola-mines | Phosphatidylethanolamine | PE | 70 |
|  |  |  | PE-O | 50 |
|  |  |  | LPE | 13 |
|  |  |  | LPE-O | 14 |
|  | Glycerophosphoinositols | Phosphatidylinositol | PI | 33 |
|  |  | Lysophosphatidylinositol | LPI | 6 |
|  |  | Ether-linked Phosphatidylinositol | PI-O | 2 |
|  | Glycerophosphoglycerols | Phosphatidylglycerol | PG | 33 |
|  |  | Lysophosphatidylglycerol | LPG | 6 |
|  | Glycerophosphoserines | Phosphatidylserine | PS | 28 |
|  |  | Lysophosphatidylserine | LPS | 9 |
|  |  | Ether-linked lysophosphatidylserine | LPS-O | 1 |
|  | Glycerophosphoglycero-phosphoglycerols | Cardiolipin | CL | 27 |
| Glycerolipids | Monoradylglycerols | Monoacylglycerol | MG | 11 |
|  | Diradylglycerols | Diacylglycerol | DG | 57 |
|  |  | alkyl-acylglycerols | DG-O | 2 |
|  | Triradylglycerols | Triacylglycerol | TG | 88 |
| Sterol Lipids | Sterol esters | Cholesterol ester | CE | 12 |
|  | Other sterols | Other Sterol | ST | 8 |

**Supplementary Table 2**. Significantly altered lipids classified by subclass and FC in the volcano plots.

| Lipid subclass | Skin tissue | | | | Epithelial tissue (FRT) | | | |
| --- | --- | --- | --- | --- | --- | --- | --- | --- |
|  | WT vs E6 | | WT vs E6/E7 | | WT vs E6 | | WT vs E6/E7 | |
|  | FC<2* | FC>2 | FC<2 | FC>2 | FC<2 | FC>2 | FC<2 | FC>2 |
| FFA | 1 | 2 | 1 | 4 |  |  | 0 | 1 |
| GlcCer | 1 | 0 | 11 | 4 | 3 | 1 | 1 | 1 |
| Cer-ADS | 2 | 0 | 4 | 0 |  |  |  |  |
| Cer-AP | 1 | 4 | 4 | 5 |  |  |  |  |
| Cer-AS | 3 | 4 | 3 | 9 |  |  |  |  |
| Cer-EOS | 0 | 1 | 1 | 0 |  |  | 1 | 0 |
| Cer-NDS | 3 | 0 | 6 | 0 |  |  |  |  |
| Cer-NP | 0 | 6 | 2 | 4 |  |  |  |  |
| Cer-NS | 1 | 3 | 3 | 5 |  |  |  |  |
| DG | 0 | 1 | 3 | 2 | 3 | 0 | 1 | 0 |
| OxFA | 3 | 19 | 3 | 22 |  |  |  |  |
| LPC | 0 | 2 | 0 | 8 | 0 | 1 | 1 | 0 |
| LPC-O | 0 | 1 | 0 | 5 |  |  | 0 | 1 |
| PC | 1 | 5 | 3 | 27 | 2 | 0 | 2 | 7 |
| PC-O | 0 | 3 | 2 | 16 |  |  | 0 | 1 |
| PE | 1 | 3 | 2 | 8 |  |  | 1 | 2 |
| PG | 0 | 1 | 1 | 7 |  |  | 0 | 1 |
| SP | 2 | 0 | 3 | 1 | 1 | 0 | 0 | 1 |
| ST | 0 | 1 | 0 | 3 | 0 | 1 |  |  |
| cGSL | 0 | 1 | 0 | 1 | 0 | 1 |  |  |
| SM | 1 | 1 | 12 | 1 |  |  |  |  |
| CAR | 0 | 1 | 0 | 8 |  |  |  |  |
| CE | 0 | 2 | 0 | 6 | 0 | 6 |  |  |
| CL |  |  | 9 | 0 | 0 | 1 | 0 | 1 |
| FA-AA |  |  | 0 | 1 |  |  |  |  |
| LPE |  |  | 0 | 2 |  |  |  |  |
| LPE-O |  |  | 0 | 1 |  |  |  |  |
| LPS |  |  | 0 | 1 |  |  |  |  |
| LPS-O |  |  | 0 | 1 |  |  |  |  |
| MG |  |  | 2 | 0 |  |  |  |  |
| PE-O |  |  | 4 | 2 |  |  |  |  |
| PI |  |  | 1 | 2 |  |  | 0 | 3 |
| PS |  |  | 2 | 2 |  |  | 0 | 2 |
| TG |  |  | 13 | 0 |  |  |  |  |
| DG-O |  |  |  |  | 1 | 0 |  |  |

Fold change = Abundance of E6/WT, or Abundance of E6E7/WT

**Supplementary Table 3.** Lipid set enrichment analysis (LSEA) of lipidomics data of skin/ear tissue samples from HPV oncogenic mouse model. Log odds ratio (LOR) of lipid subclasses with FDR<0.05 are reported.

| Genotype | TG | SM | Cer-ADS | Cer-NDS | CL | GlcCer | LPG | CE | LPC | CAR | OxFA | PC |
| --- | --- | --- | --- | --- | --- | --- | --- | --- | --- | --- | --- | --- |
| E6 vs WT | -0.8 | -0.5 | -1.8 | -1.0 | NS* | NS | -1.7 | NS | NS | NS | 1.1 | NS |
| E6/E7 vs WT | -0.8 | -1.0 | -2.0 | -1.1 | -0.8 | -0.7 | NS | 0.8 | 0.6 | 0.7 | 0.9 | 0.4 |

*NS: not significant.

**Supplementary Table 4.** Lipid set enrichment analysis (LSEA) of lipidomics data of epithelial FRT tissue samples from HPV oncogenic mouse model. Log odds ratio (LOR) of lipid subclasses with FDR<0.05 are reported.

| Genotype | TG | DG | GlcCer | CAR | PE-O | LPC-O | PC-O | PE | PG | PI | PC | Cer-AS | LPE-O | CE | LPC |
| --- | --- | --- | --- | --- | --- | --- | --- | --- | --- | --- | --- | --- | --- | --- | --- |
| E6 vs WT | -1.0 | -0.7 | -0.7 | -0.6 | 0.4 | 0.8 | 0.9 | 0.4 | 0.6 | 0.6 | 0.3 | NS | NS | 1.7 | NS |
| E6/E7 vs WT | -1.4 | -0.6 | NS* | NS | NS | 1.0 | 0.5 | NS | NS | 0.6 | NS | 0.4 | 1.4 | 1.1 | 0.5 |

*NS: not significant.
